# *Escherichia coli* BarA-UvrY regulates the *pks* island and kills Staphylococci via the genotoxin colibactin during interspecies competition

**DOI:** 10.1371/journal.ppat.1010766

**Published:** 2022-09-06

**Authors:** Jun Jie Wong, Foo Kiong Ho, Pei Yi Choo, Kelvin K. L. Chong, Chee Meng Benjamin Ho, Ramesh Neelakandan, Damien Keogh, Timothy Barkham, John Chen, Chuan Fa Liu, Kimberly A. Kline

**Affiliations:** 1 Singapore Centre for Environmental Life Sciences Engineering, Nanyang Technological University, Singapore, Singapore; 2 Singapore Centre for Environmental Life Sciences Engineering, Interdisciplinary Graduate Programme, Nanyang Technological University, Singapore, Singapore; 3 Nanyang Technological University Institute for Health Technologies, Interdisciplinary Graduate School, Nanyang Technological University, Singapore, Singapore; 4 School of Mechanical and Aerospace Engineering, Nanyang Technological University, Singapore, Singapore; 5 School of Biological Sciences, Nanyang Technological University, Singapore, Singapore; 6 Department of Microbiology and Immunology, Yong Loo Lin School of Medicine, National University of Singapore, Singapore, Singapore; 7 Department of Laboratory Medicine, Tan Tock Seng Hospital, Singapore, Singapore; 8 Department of Microbiology and Molecular Medicine, University of Geneva, Geneva, Switzerland; University of Tubingen, GERMANY

## Abstract

Wound infections are often polymicrobial in nature, biofilm associated and therefore tolerant to antibiotic therapy, and associated with delayed healing. *Escherichia coli* and *Staphylococcus aureus* are among the most frequently cultured pathogens from wound infections. However, little is known about the frequency or consequence of *E*. *coli* and *S*. *aureus* polymicrobial interactions during wound infections. Here we show that *E*. *coli* kills Staphylococci, including *S*. *aureus*, both *in vitro* and in a mouse excisional wound model via the genotoxin, colibactin. Colibactin biosynthesis is encoded by the *pks* locus, which we identified in nearly 30% of human *E*. *coli* wound infection isolates. While it is not clear how colibactin is released from *E*. *coli* or how it penetrates target cells, we found that the colibactin intermediate N-myristoyl-D-Asn (NMDA) disrupts the *S*. *aureus* membrane. We also show that the BarA-UvrY two component system (TCS) senses the environment created during *E*. *coli* and *S*. *aureus* mixed species interaction, leading to upregulation of *pks* island genes. Further, we show that BarA-UvrY acts via the carbon storage global regulatory (Csr) system to control *pks* expression. Together, our data demonstrate the role of colibactin in interspecies competition and show that it is regulated by BarA-UvrY TCS during interspecies competition.

## Author summary

Wound infections are often polymicrobial in nature and are associated with poor disease prognoses. *Escherichia coli* and *Staphylococcus aureus* are among the top five most cultured pathogens from wound infections. However, little is known about the polymicrobial interactions between *E*. *coli* and *S*. *aureus* during wound infections. In this study, we show that *E*. *coli* kills *S*. *aureus* both *in vitro* and in a mouse excisional wound model via the genotoxin, colibactin. We also show that the BarA-UvrY two component system (TCS) regulates the *pks* island during this mixed species interaction, acting through the carbon storage global regulatory (Csr) system to control colibactin production. Together, our data demonstrate the role of colibactin in interspecies competition and show that it is regulated by BarA-UvrY TCS during interspecies competition.

## Introduction

Chronic wound infections are often biofilm-associated and polymicrobial in nature [1–4]. Polymicrobial wound infections are associated with heightened inflammation and delayed wound healing as compared to monomicrobial wound infections [5, 6]. Within polymicrobial communities, interspecies interactions can increase the pathogenicity of either or both species, inducing virulence gene expression, enhancing growth, or promoting antibiotic tolerance and immune evasion [7–10]. Polymicrobial interactions can also be antagonistic via outcompetition for critical nutrients, by interfering with quorum sensing of competitors [11–13] or by producing antimicrobial agents to kill competitors [14–16]. Antimicrobial agents involved in competitor outcompetition include secreted bacteriocins and effector toxins, which are delivered via specialized secretion systems [14–16].

The most frequently cultured bacterial species from wound infections include *Staphylococcus aureus*, *Pseudomonas aeruginosa*, *Enterococcus spp*., *Escherichia coli*, and *Klebsiella pneumoniae* [17, 18]. The mechanistic basis of polymicrobial interactions in wounds has been examined for *S*. *aureus* together with *P*. *aeruginosa* and *Enterococcus faecalis* [7, 10, 19]. However, despite the fact that *E*. *coli* and *S*. *aureus* are among the top five most prevalent pathogens in often polymicrobial surgical, diabetic and non-diabetic wound infections [17, 18, 20, 21], and coexist within diabetic wound microbiomes [22–24], polymicrobial interaction studies between these organisms, or of *E*. *coli* within wound infections in general, are scarce. We have previously shown that *E*. *faecalis* promotes *E*. *coli* biofilm growth and virulence *in vitro* and in a mouse excisional wound infection model [25]. However, the mechanistic basis of interactions between *S*. *aureus* and *E*. *coli* remains largely unknown.

In this study, we show that *E*. *coli* antagonizes *S*. *aureus* in biofilms and planktonic growth. Both the *E*. *coli pks* island and the BarA-UvrY two component system (TCS) are required for killing *S*. *aureus*. The *pks* island encodes the biosynthetic machinery to produce colibactin, a genotoxin that causes DNA damage in eukaryotic cells and is associated with human colorectal cancer [26, 27]. Here we show that *E*. *coli* colibactin kills *S*. *aureus* by causing irreparable DNA damage. *E*. *coli* also antagonizes the growth and survival of *S*. *aureus* upon co-infection in a mouse excisional wound model, and this antagonism is dependent on the *pks* island and the BarA-UvrY TCS. Finally, we show that the BarA-UvrY TCS regulates the expression of the *pks* island through the Csr system during interspecies competition. Taken together, our data demonstrate the mechanism by which *E*. *coli* colibactin acts in interspecies competition to kill *S*. *aureus* during wound infection.

## Results

### *E*. *coli* antagonizes the growth of *Staphylococcus* species during *in vitro* co-culture

**Fig 1:**
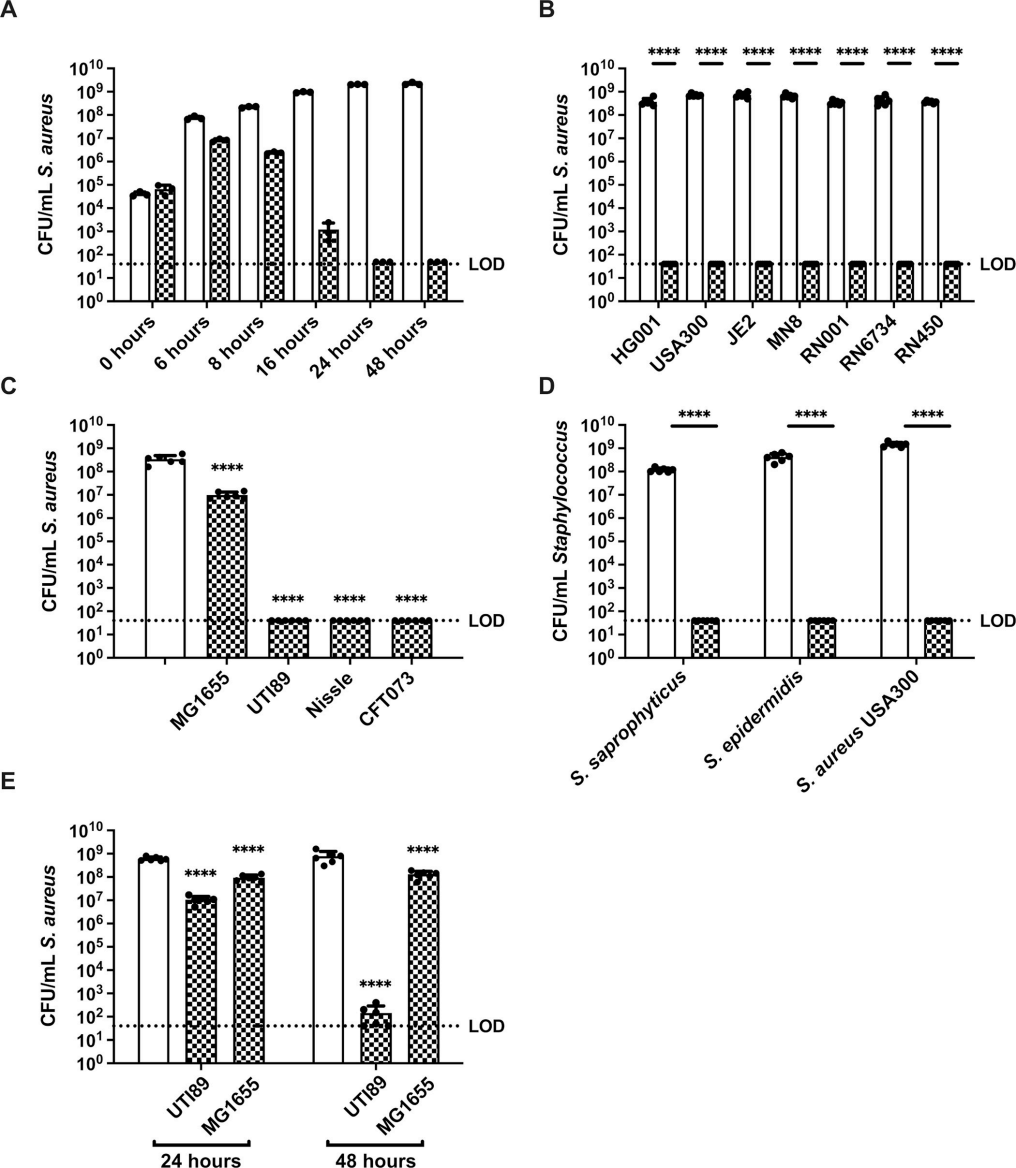
*E*. *coli* kills *Staphylococcus spp in vitro*. **(A)** Enumeration of *S*. *aureus* USA300 grown alone or co-cultured with *E*. *coli* UTI89 in macrocolonies for 0, 6, 8, 16, 24 and 48 hours. N = 3 independent experiments. **(B)** Enumeration of different strains of *S*. *aureus* grown alone or together with *E*. *coli* UTI89 in macrocolonies for 24 h. N = 6 independent experiments. **(C)** Enumeration of *S*. *aureus* HG001 from single species or mixed species macrocolonies containing the indicated strain of *E*. *coli*, at 24 h. N = 6 independent experiments. Statistical significance was determined by one-way ANOVA with Dunnett’s test for multiple comparison. **(D)** *Staphylococci* from single species or mixed species macrocolonies co-cultured with *E*. *coli* UTI89 for 24 h. N = 6 independent biological experiments. **(E)** Enumeration of *S*. *aureus* after planktonic growth alone or mixed with either *E*. *coli* UTI89 or MG1655 for 24 or 48 hours. N = 6 independent experiments (**A-E**) Data from single species macrocolonies or planktonic culture are indicated with open bars, and data from mixed species (all inoculated at a ratio of 1_EC_:1_SA_) macrocolonies are indicated with checked bars. Individual data points from each biological replicate are indicated with closed circles. (**B, D, E**) Statistical significance was determined by two-way ANOVA with Sidak’s (A and C) and Tukey’s (D) test for multiple comparisons. ****p< 0.0001. Error bars represent SD from the mean. All statistical tests were performed on log-transformed CFU data. (See **S1 Fig** for paired *E*. *coli* CFU to match the *S*. *aureus* data shown here).

To investigate the mechanistic basis of interactions between *S*. *aureus* and *E*. *coli*, we assessed the growth of each species within macrocolony biofilms and planktonic co-culture, followed by enumeration of viable CFU of each species on selective media. We first grew *E*. *coli* UTI89 and *S*. *aureus* USA300 dual species macrocolonies and enumerated CFU over time. While *S*. *aureus* CFU within single species macrocolonies increased between zero and 24 hours, *S*. *aureus* CFU from dual species macrocolonies started to fall at around 6 hours and fell below the limit of detection by 24 hours (**Fig 1A**), and *E*. *coli* CFU were unaffected by the presence of *S*. *aureus* (**S1A Fig**). We thus used the 24 hour time point for subsequent experiments unless otherwise indicated. While *E*. *coli* UTI89 is a uropathogenic strain [28], it is able to establish an infection in a murine wound model [25], and both *E*. *coli* wound and uropathogenic isolates share similar virulence profiles and are most frequently of the B2 phylogenetic group [29, 30]. Next, we grew dual species macrocolonies of *E*. *coli* UTI89 partnered with one of six different strains of *S*. *aureus*. By 24 hours, the CFU of all *S*. *aureus* strains tested fell from the initial inoculum of 1 x 10^5^ CFU to below limit of detection in the presence of *E*. *coli*, demonstrating that *E*. *coli* UTI89 can kill *S*. *aureus* within macrocolony co-culture (**Fig 1B**). Conversely, not all *E*. *coli* strains tested could kill *S*. *aureus* strain HG001; *E*. *coli* MG1655 did not kill *S*. *aureus*, suggesting that this phenotype is specific to certain *E*. *coli* strains (**Fig 1C**). To test whether *E*. *coli* could similarly kill other members of the Staphylococcus genus, we grew macrocolonies of *E*. *coli* with *S*. *saprophyticus* and *S*. *epidermidis* and observed that both *Staphylococcus* species were killed (**Fig 1D**). We also observed *S*. *aureus* killing in planktonic culture with *E*. *coli*; however, the killing was less efficient and took 48 hours to approach the limit of detection (**Fig 1E**). Similar to the macrocolony assay, *E*. *coli* MG1655 was also unable to kill *S*. *aureus* during planktonic growth (**Fig 1E**). *E*. *coli* CFU remained unchanged when grown alone or co-cultured with *S*. *aureus* (**S1B–S1E Fig**). These results show that *E*. *coli* can kill *S*. *aureus* both within macrocolony biofilms and planktonic cultures.

### *E*. *coli* does not kill *S*. *aureus* by prophage induction

**Fig 2:**
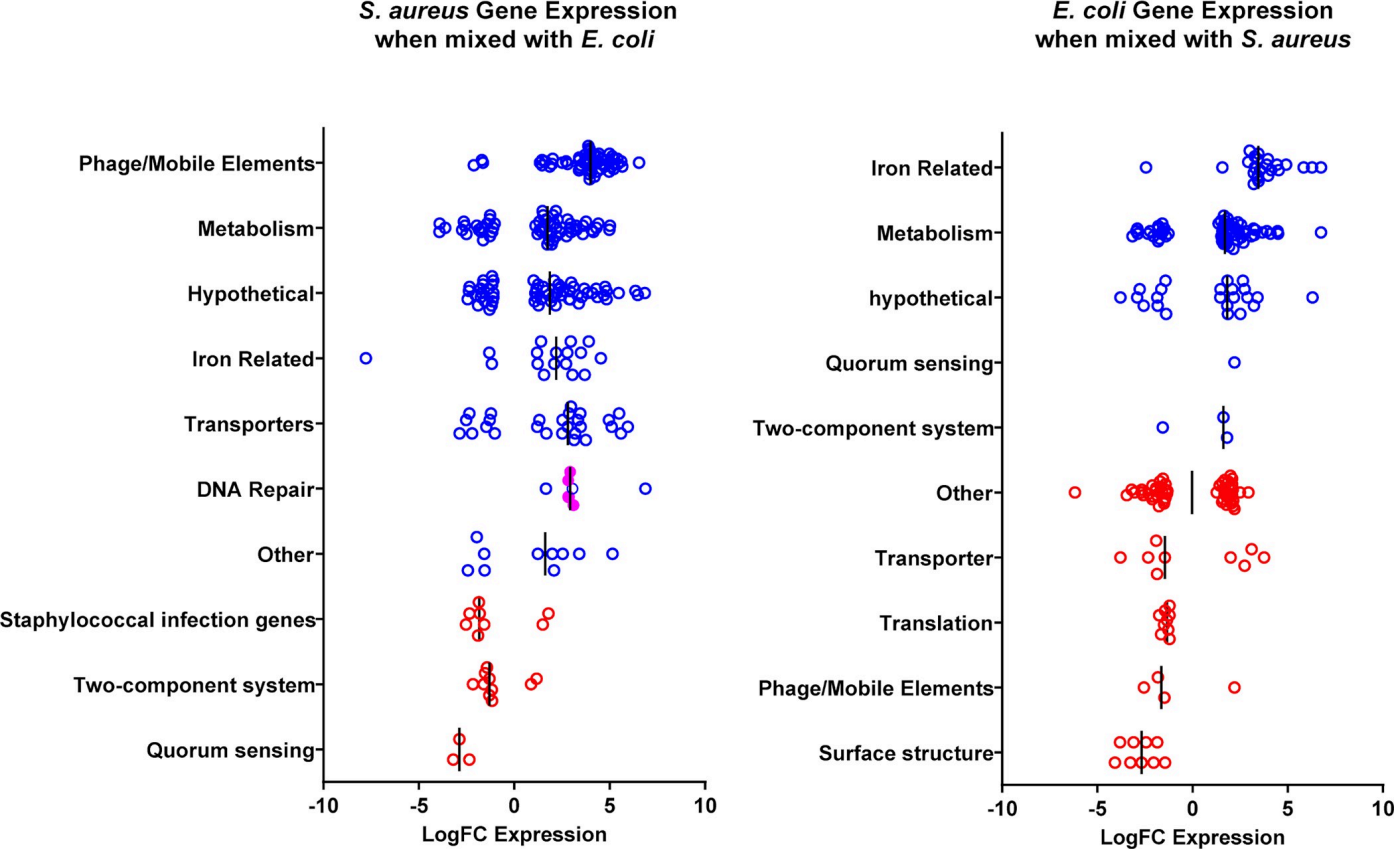
Co-culture of *E*. *coli* and *S*. *aureus* promotes differential gene expression. Transcription comparison between single species *S*. *aureus* strain HG001 or *E*. *coli* strain UTI89 macrocolonies and mixed species macrocolonies (1EC:1SA) after 6 h incubation, a time point at which sufficient live *S*. *aureus* could be recovered. Vertical black lines represent median values for each gene category. Each circle represents a gene that is differentially regulated (p<0.05, FDR<0.05) in the mixed species macrocolony compared to the single species macrocolony in the respective functional categories, with blue color indicating a functional category where the median value shows increased expression in the mixed species macrocolony and red color indicating decreased expression. Closed magenta circles represents *recA* and *uvrAB* genes. Data represent ≥2 biological replicates.

To gain insight into the mechanism underlying *E*. *coli*-mediated killing of *S*. *aureus*, we examined the gene expression profiles of both *E*. *coli* and *S*. *aureus*, comparing single and mixed species macrocolonies. We extracted RNA from macrocolonies grown for 6 hours since viable *S*. *aureus* could be recovered at the timepoint (**Fig 1A**). Several gene expression pattern changes were distinctive **(Fig 2 and S1 and S2 Tables)**. First, genes related to iron acquisition and utilization were induced in both species within mixed species macrocolonies compared to single species macrocolonies, suggesting that iron is limiting during co-culture. Second, genes associated with phage or mobile elements comprised the most highly induced functional category for *S*. *aureus* within mixed species macrocolonies. In *S*. *aureus*, the DNA damage-induced SOS response can induce resident prophages, leading to *S*. *aureus* lysis [31]. Since we also observed that genes involved in DNA repair, such as *recA* and *uvrA*, were upregulated in *S*. *aureus* mixed species macrocolonies, we hypothesized that prophage induction contributes to *E*. *coli*-mediated killing of *S*. *aureus* as recently suggested for some *S*. *aureus* prophage-expressing strains [32]. However, when we tested the prophage-cured *S*. *aureus* strain RN450 [33] in the mixed species macrocolony assay, we observed that it was also readily killed by *E*. *coli* (**Fig 1B**), indicating that prophage induction was not the mechanism by which *E*. *coli*-induced death in *S*. *aureus* in this setting.

### The colibactin *pks* island and BarA-UvrY TCS contributes to *E*. *coli*-mediated growth antagonism of *S*. *aureus*

**Fig 3:**
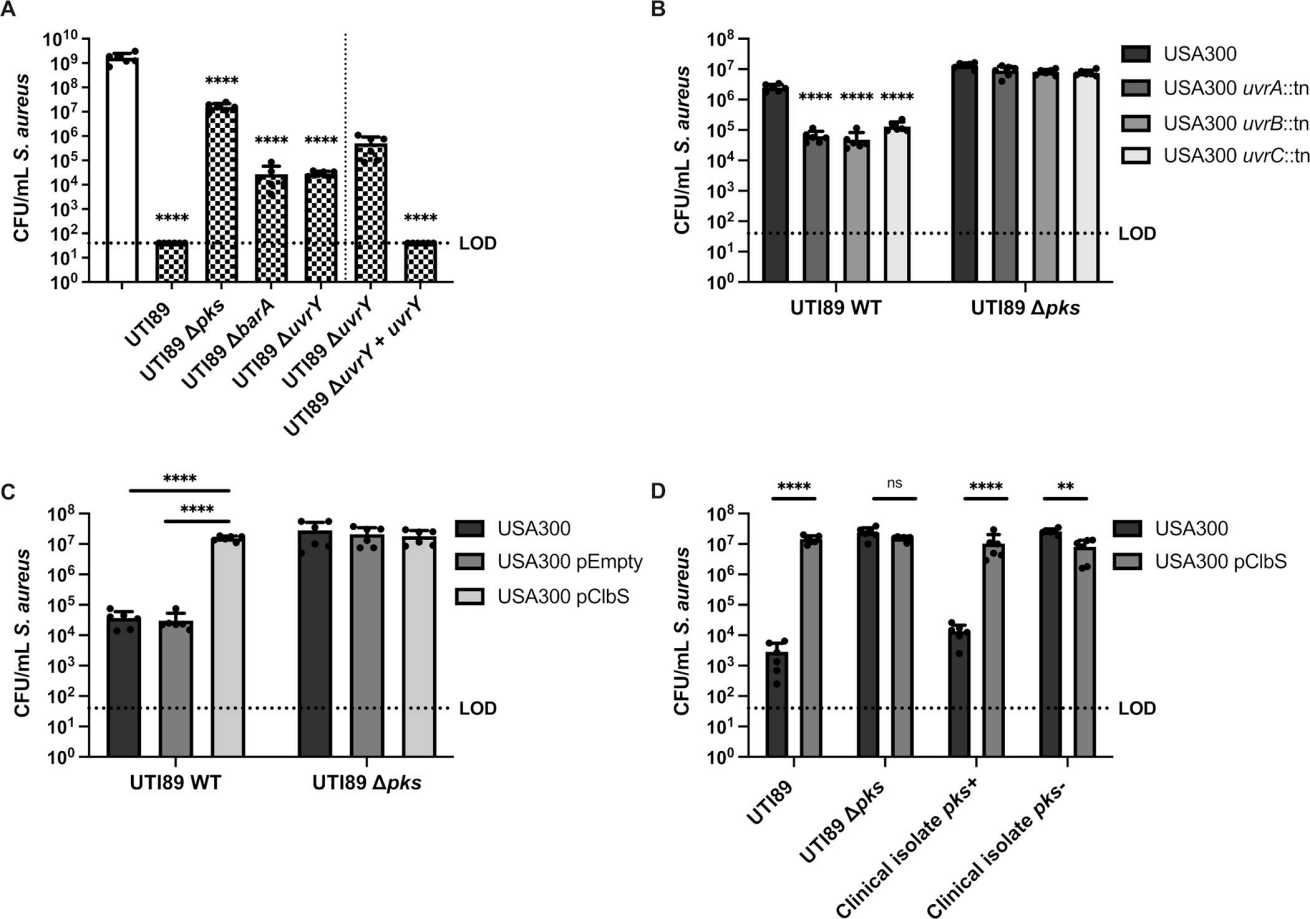
The BarA-UvrY two component system (TCS) and the *pks* island are required for *E*. *coli*-mediated killing of *S*. *aureus*. **(A)** Enumeration of *S*. *aureus* USA300 LAC and mixed (1_EC_:1_SA_) macrocolonies with either UTI89 wild type, knockout mutants of the *pks* island, *barA* or *uvrY* or complemented strains. The vertical dotted line indicates that data collected on either side were collected from separate experiments. Data from single species macrocolonies are indicated with open bars, and data from mixed species macrocolonies are indicated with checked bars. N = 6 independent biological experiments. Statistical significance was determined by one-way ANOVA with Dunnett’s test for multiple comparison. **(B)** Enumeration of *S*. *aureus* USA300 LAC from 8 h macrocolonies. Wild type *S*. *aureus* USA300 LAC and *uvrABC* transposon mutants were mixed 1_EC_:1_SA_ with either *E*. *coli* UTI89 or knockout mutants of the *pks* island. N = 6 independent experiments. **(C)** Enumeration of *S*. *aureus* from 24 h macrocolonies. Wild type *S*. *aureus* USA300 LAC was transformed with pJC-2343 (pEmpty) or pJC-2343-ClbS (pClbS) and mixed 1:1 with either *E*. *coli* UTI89 or knockout mutants of the *pks* island. N = 6 independent experiments. Individual data points are indicated with closed circles. **(D)** Enumeration of *S*. *aureus* from 24 h macrocolonies. Wild type *S*. *aureus* USA300 LAC or USA300 pClbS was mixed 1:1 with either *E*. *coli* UTI89, UTI89 Δ*pks* island, *pks*+ or *pks*- clinical isolate. N = 6 independent experiments. **(B-D)** Statistical significance was determined by two-way ANOVA with Tukey’s test for multiple comparisons. ****p< 0.0001, error bars represent SD from the mean. All statistical tests were performed on log-transformed CFU data.

To identify the genes involved in *E*. *coli*-mediated killing of S. *aureus*, we screened 14,828 *E*. *coli* UTI89 transposon mutants for failure to antagonize *S*. *aureus* growth in a macrocolony assay. We validated each *E*. *coli* gene identified in the transposon screen for its inability to kill *S*. *aureus* after 24 hours of macrocolony co-culture and the transposon insertion sites for validated mutants were determined by whole genome sequencing. The majority (99 out of 108) of transposon insertions mapped to genes of the *pks* island and genes encoding the two-component system (TCS) BarA-UvrY (**S3 Table**). To confirm that these *E*. *coli* loci impacted *S*. *aureus* survival, we generated deletion mutants comprising the entire *pks* island, as well as for *barA* and *uvrY*, all of which had significantly higher *S*. *aureus* CFU in the mixed species macrocolony compared to *E*. *coli* UTI89 wild type (**Fig 3A**). These mutants grew as well as wild type (**S2 Fig**), showing that attenuation of the killing phenotype was not due to growth differences. Although co-culture with *E*. *coli* Δ*pks* did not restore *S*. *aureus* growth to single species growth levels, *S*. *aureus* CFU were similar to that in co-culture with *E*. *coli* MG1655 (**Fig 1C**) which does not possess the *pks* island [34, 35] and which also failed to kill *S*. *aureus*. We therefore surmise that nutrient competition within mixed species macrocolonies results in a 1–2 log decrease in *S*. *aureus* compared to *S*. *aureus* grown alone. Notably, co-culture with either Δ*barA* or Δ*uvrY* only partially restored *S*. *aureus* growth, suggesting that the growth antagonism is not completely abolished when the BarA-UvrY TCS is inactivated (**Fig 3A**). Chromosomal complementation of *uvrY* in the Δ*uvrY* mutant, restores the killing phenotype. These data show that the *pks* island is necessary for *S*. *aureus* killing and suggest that the BarA-UvrY TCS may either directly or indirectly regulate the expression of the *pks* island or the activity of its gene products.

### The genotoxin colibactin kills *S*. *aureus* by inducing DNA damage

The *pks* island encodes enzymes required for the synthesis of the genotoxin colibactin [36]. *E. coli* strains carrying the 54 kb *pks* island generate DNA adducts and induce DNA crosslinks in mammalian cells [26, 37–40]. In bacteria, DNA adducts and crosslinks can be repaired via the nucleotide excision repair (NER) pathway, facilitated by the UvrABC endonuclease complex [41]. Accordingly, *pks+ E*. *coli* strains lacking both UvrB and the ClbS colibactin resistance protein, which protects *E*. *coli* from colibactin-mediated autotoxicity, are severely impaired for growth [42]. Consistent with DNA damage, we found that *S*. *aureus uvrAB* genes were significantly upregulated in mixed species macrocolonies with *E*. *coli* (**Fig 2**). Accordingly, we predicted that disruption of the *S*. *aureus* NER pathway would accelerate its killing by *E*. *coli*. We thus examined CFU following macrocolony co-culture of *S*. *aureus uvrA*, *uvrB* and *uvrC* transposon mutants with *E*. *coli* at 8 hours, a timepoint prior to complete *S*. *aureus* eradication, in order to detect differences between wild type *S*. *aureus* and *uvrABC* mutants, and observed accelerated *pks*-dependent killing and significantly fewer *S*. *aureus* CFU suggesting a role for NER in the protection of *S*. *aureus* from colibactin-mediated killing **(Fig 3B)**. To further investigate the role of colibactin in *S*. *aureus* killing, we expressed the colibactin resistance protein ClbS in *S*. *aureus*. Expression of ClbS in *S*. *aureus* cells conferred full protection from *E*. *coli pks*-mediated killing, suggesting that colibactin is responsible for *S*. *aureus* cytotoxicity **(Fig 3C)**. To determine the prevalence of *pks* island in *E*. *coli* isolated from human wound swabs, we screened 58 isolates for presence of *pks* by PCR flanking the *clbB* gene and found 17 out of the 58 clinical isolates (29.3%) were *pks* positive (*pks*+) (**S4 Table**). We then randomly picked ten of each *pks*+ and *pks*- *E*. *coli* clinical isolates to perform co-culture macrocolony assay with *S*. *aureus*. We found 9 of 10 *pks+* clinical isolates were able to kill *S*. *aureus*, while no *pks*- clinical isolates killed *S*. *aureus* (**S3 Fig**). Finally, we show that expression of ClbS in *S*. *aureus* conferred protection against killing by a *pks*+ *E*. *coli* clinical isolate (**Fig 3D**) comparable to *S*. *aureus* CFU in co-culture with a *pks*- *E*. *coli* isolate. Collectively, these data support the interpretation that killing of *S*. *aureus* by colibactin-producing *E*. *coli* is mediated by DNA damage, which is consistent with the known genotoxic effects of colibactin.

### *N*-myristoyl-D-Asn causes pore formation in *S*. *aureus*

Maturation of colibactin requires the removal of the prodrug motif, *N*-myristoyl-D-Asn (NMDA), by ClbP peptidase [43, 44]. NMDA is the most abundant of the *pks* island metabolites along with its analogues that vary in acyl chain lengths (C_12_ to C_16_) [45]. These intermediates do not exhibit cytotoxic or genotoxic activity in HeLa cells; however, NMDA can modestly inhibit *Bacillus subtilis* growth [45]. Thus, we investigated if the production of NMDA could provide an alternative explanation for the killing of *S*. *aureus* by *pks*+ *E*. *coli*. We synthesized NMDA and added it at increasing concentrations to *S*. *aureus* but we observed minimal dose-dependent growth inhibition, and only at high concentrations of 600 μM (**S4A Fig**). Despite the absence of significant toxicity, NMDA treatment resulted in increased *S*. *aureus* membrane permeability as measured by propidium iodide uptake using flow cytometry (**S4B Fig**). Thus, NMDA, which is the most abundant colibactin metabolite isolated from culture supernatants [45] and is therefore likely released from *E*. *coli* along with colibactin, can compromise *S*. *aureus* membrane integrity.

### BarA-UvrY TCS regulates *pks* island genes via the Csr system during interspecies competition

**Fig 4:**
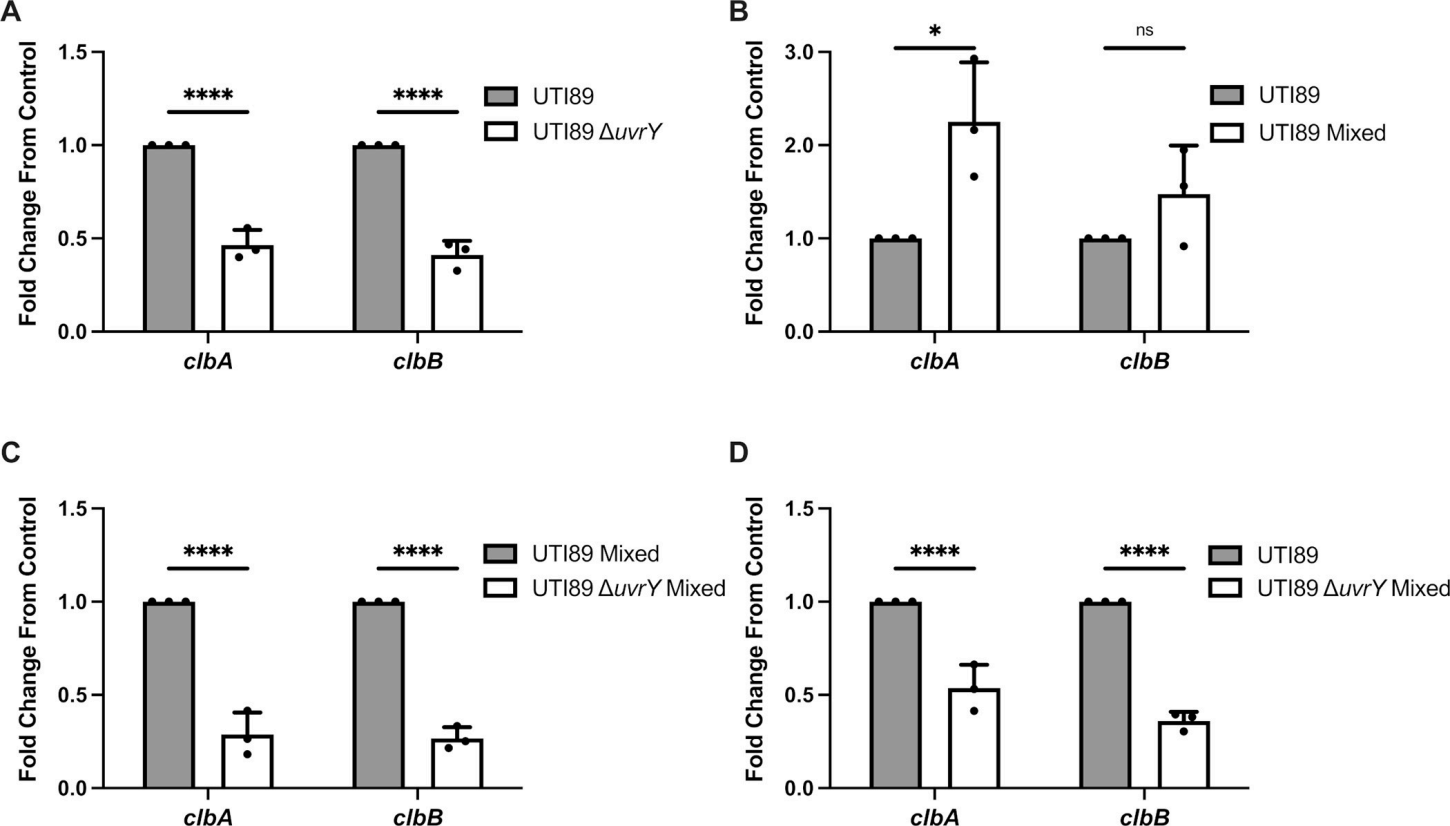
Co-culture of *E*. *coli* and *S*. *aureus* induces *pks* island expression in a BarA-UvrY TCS dependent manner. **(A)** RT-qPCR of *E*. *coli* single species macrocolonies and *E*. *coli* Δ*uvrY* single species macrocolonies at 24 h. **(B)** RT-qPCR of *E*. *coli* single species macrocolonies and *E*. *coli* mixed species macrocolonies at 24 h. **(C)** RT-qPCR of *E*. *coli* mixed species macrocolonies and *E*. *coli* Δ*uvrY* mixed species macrocolonies at 24 h. **(D)** RT-qPCR of *E*. *coli* single species macrocolonies and *E*. *coli* Δ*uvrY* mixed species macrocolonies at 24 h. N = 3 independent experiments, each the average of 4 technical replicates. Gene expression was normalized to the *gyrA* housekeeping gene. Individual data points from each biological replicate are indicated with closed circles. Statistical significance was determined by Bonferroni’s multiple comparisons test for two-way ANOVA, ****p< 0.0001, error bars represent SD from the mean.

In *E*. *coli*, *pks* island genes are upregulated when iron is limited in a Fur-dependent manner, while *pks* island genes are downregulated when iron is in abundance [46, 47]. While gene expression profiling indicates that both *E*. *coli* and *S*. *aureus* are experiencing iron-limitation in mixed species macrocolonies, iron-supplementation experiments did not prevent *E*. *coli*-mediated killing of *S*. *aureus* (**S5 Fig**), suggesting that iron restriction is not the sole driver of *pks* expression in this mixed species interaction. The BarA-UvrY TCS have been recently shown to be involved in the regulation of the *pks* island [48]. Since both *pks* and *barA/uvrY E*. *coli* mutants failed to kill *S*. *aureus*, we hypothesized that the BarA-UvrY TCS is involved in the regulation of the *pks* island during interspecies competition. To investigate this, we first compared the expression of *pks* island genes (*clbA* and *clbB*) between single species macrocolonies of wild type *E*. *coli* and *E*. *coli* Δ*uvrY* and found out that the expression of both *clbA* and *clbB* were significantly lower in the *E*. *coli* Δ*uvrY* macrocolony (**Fig 4A**). Next, we examined the expression of *pks* island genes in the presence or absence of *S*. *aureus*, and found that *E*. *coli clbA* expression was significantly increased in mixed species macrocolonies compared to *E*. *coli* single species macrocolonies (**Fig 4B)**. By contrast, expression of both *clbA* and *clbB* were significantly lower when *E*. *coli* Δ*uvrY* was co-cultured in macrocolonies with *S*. *aureus*, compared to wild type *E*. *coli*-*S*. *aureus* macrocolonies, suggesting that the BarA-UvrY TCS is involved in regulating *pks* island gene expression (**Fig 4C**). Moreover, *S*. *aureus*-dependent induction of *clbA* and *clbB* gene expression in wild type *E*. *coli* was significantly attenuated upon macrocolony co-culture with *E*. *coli* Δ*uvrY* (**Fig 4D**), together suggesting that *E*. *coli* can use the BarA-UvrY system to sense *S*. *aureus* and induce *pks* island (*clbA* and *clbB*) gene expression.

**Fig 5:**
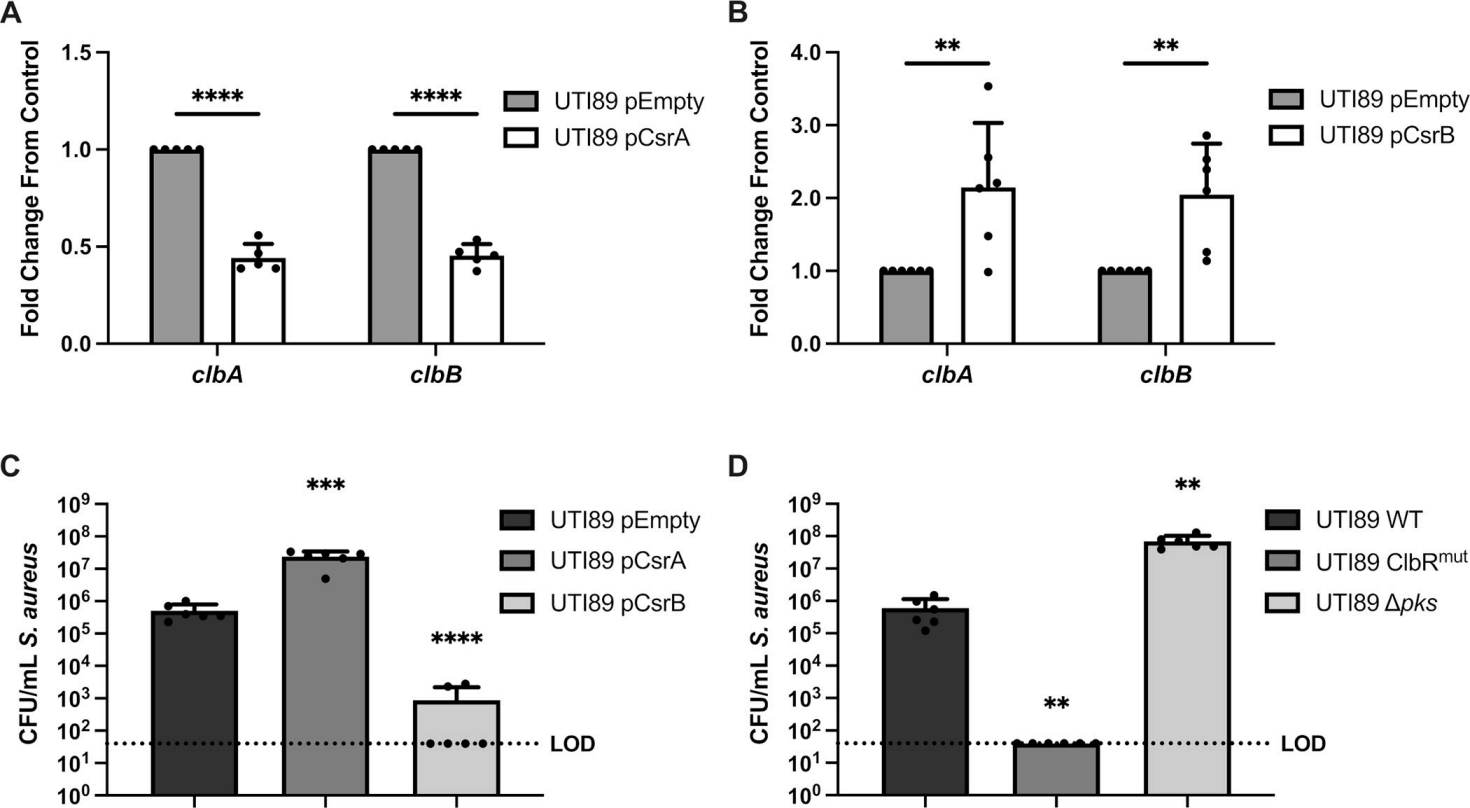
The BarA-UvrY TCS regulates the *pks* island via the Csr system. **(A)** RT-qPCR of 16 h macrocolonies of *E*. *coli* pTrc99a (pEmpty) and *E*. *coli* pTrc99a-CsrA (pCsrA). **(B)** RT-qPCR of 16 h macrocolonies of *E*. *coli* pEmpty and *E*. *coli* pCsrB. N = 5–6 independent experiments, each the average of 2 technical replicates. Gene expression was normalized to the *gyrA* housekeeping gene. Statistical significance was determined by two-way ANOVA with Bonferroni’s test for multiple comparison, **p< 0.01, ***<p< 0.001, ****p< 0.0001, error bars represent SD from the mean. **(C)** Enumeration of *S*. *aureus* from 16 h mixed macrocolonies (1_EC_:1_SA_) with either *E*. *coli* pEmpty, *E*. *coli* pCsrA or *E*. *coli* pCsrB. N = 6 independent experiments. (**D**) Enumeration of *S*. *aureus* mixed macrocolonies (1_EC_:1_SA_) from 16 h with either *E*. *coli* WT, *E*. *coli* ClbR^mut^ or *E*. *coli pks* deletion mutant. N = 6 independent experiments. Individual data points are indicated with closed circles. (**C and D**) Statistical significance was determined by Ordinary One-way ANOVA with Dunnett’s test for multiple comparison. ****p< 0.0001, error bars represent SD from the mean. Statistical tests were performed on log-transformed data.

The BarA-UvrY TCS regulates the expression of the Csr system, which in turn regulates a variety of metabolic and virulence genes via the global regulator CsrA [49]. CsrA is a post-transcriptional regulator that can either promote or repress gene expression [50]. Activation of the BarA-UvrY TCS, leads to the expression of the sRNAs CsrB and CsrC, which bind to CsrA and inhibit the regulatory activity of CsrA [50]. Therefore, if BarA-UvrY regulates *pks* transcription via CsrA, we hypothesized that increasing expression of CsrA would repress *pks* island gene expression and increasing expression of CsrB would lead to the upregulation of *pks* island genes. Consistent with these predictions, we examined 16 hour macrocolonies in which we observed a steep drop in *S*. *aureus* CFU (**Fig 1A**) and predicted substantial *pks* gene expression, and observed that overexpression of CsrA leads to the downregulation of *pks* island genes, suggesting that CsrA is a negative regulator of the *pks* island (**Fig 5A**). Conversely, overexpression of CsrB resulted in upregulation of *pks* island genes (**Fig 5B**). Co-culturing the *E*. *coli* overexpression strains with *S*. *aureus* in macrocolonies was consistent with the *pks* expression data, such that CsrA overexpression led to reduced *pks* gene expression and *S*. *aureus* killing, and CsrB overexpression led to increased *pks* gene expression and enhanced killing (**Fig 5C**). CsrA regulates gene expression by binding to its target mRNA at GGA motifs, which can be found in the 5’ untranslated region, early coding region, and the stem-loop structure of the mRNA [51–53]. ClbR is a transcriptional regulator of the *pks* locus [54]. To test whether CsrA regulates ClbR via interaction with *clbR* mRNA GGA motifs, we generated a *E*. *coli* strain where the GGA motifs in *clbR* were modified (**S5 Fig**), which we predicted would reduce CsrA binding efficiency to *clbR* mRNA, as has been reported for other CsrA mRNA substrates [49, 52]. We hypothesized that this strain, *E*. *coli clbR*^mut^, would significantly increase *S*. *aureus* killing because CsrA repression of *clbR* expression would be alleviated due to reduced CsrA binding to *clbR* mRNA. Consistent with this hypothesis, we observed that *E*. *coli clbR*^mut^ kills *S*. *aureus* faster than wild type *E*. *coli*, as expected as a result of *pks* derepression and increased colibactin synthesis (**Fig 5D**). Collectively, these data demonstrate that the *E*. *coli* BarA-UvrY TCS senses *S*. *aureus* and responds by inducing *pks* gene expression (*clbA* and *clbB*) via CsrA which acts as a negative regulator of the *pks* island.

### *E*. *coli* antagonizes the growth of *S*. *aureus* in a mouse model of wound infection

**Fig 6:**
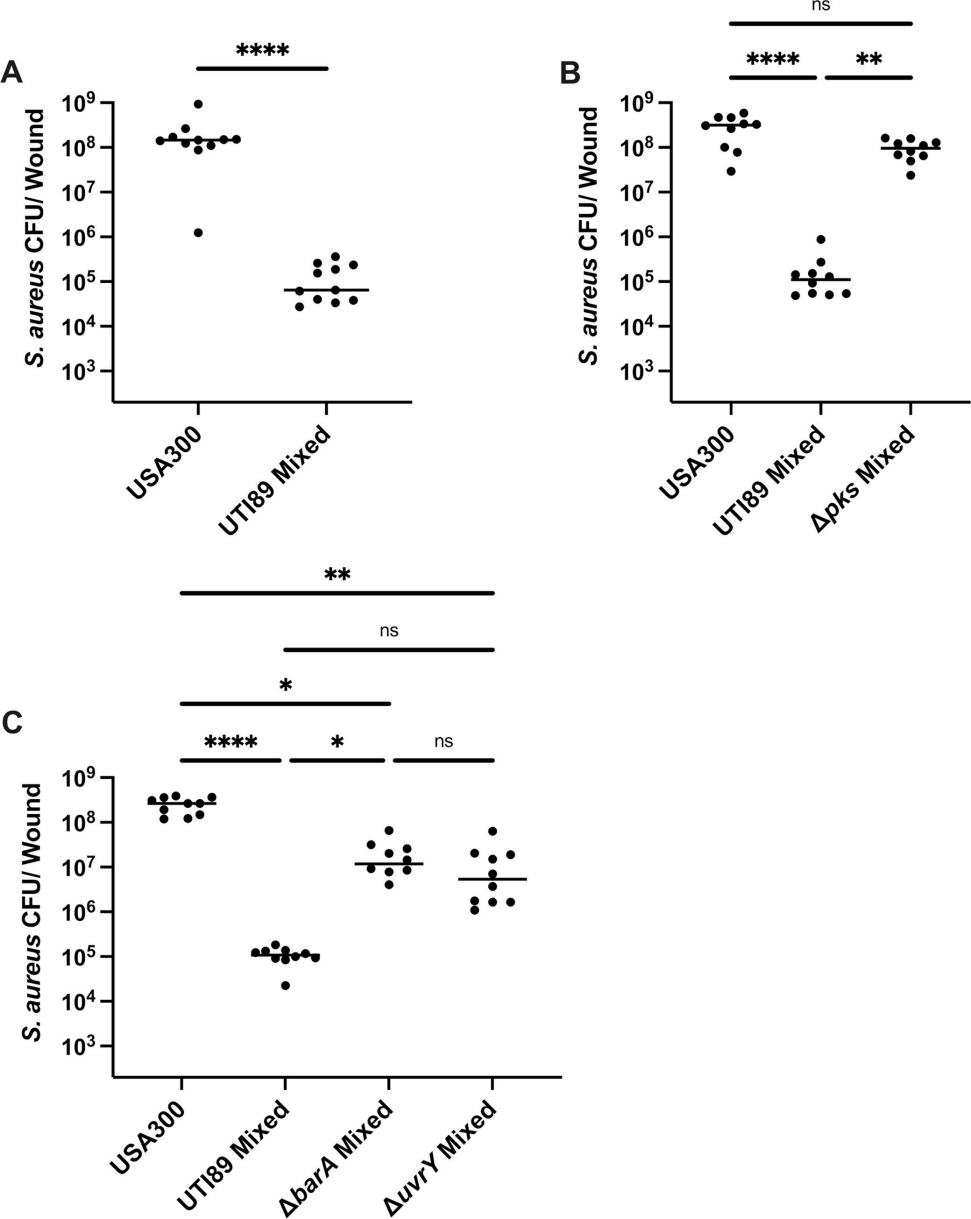
*E*. *coli* antagonizes *S*. *aureus* growth during wound infection and antagonism is dependent on the *pks* island and the BarA-UvrY TCS. Mice were co-infected with *E*. *coli* UTI89 and *S*. *aureus* USA300 LAC at 1–2 x 10^6^ CFU/wound. Wound CFU were enumerated at 24 h post infection. *S*. *aureus* single species infection or co-infection with **(A)** *E*. *coli* UTI89 WT, **(B)** *E*. *coli pks* mutant, or **(C)** BarA-UvrY TCS mutants. Each black circle represents one mouse, horizontal lines represent the median. N = 2 independent experiments, each with 5–6 mice per group. Statistical analysis was performed using Kruskal-Wallis test with Dunn’s post-test to correct for multiple comparisons. *p< 0.05, **p< 0.01, ****p< 0.0001. (See **S6 Fig** for paired *E*. *coli* CFU to match the *S*. *aureus* data shown here).

To determine whether *E*. *coli* could similarly antagonize *S*. *aureus* growth *in vivo* within a mixed species wound infection, we infected excisional wounds of C57BL/6 mice with 10^6^ CFU each of *E*. *coli* and *S*. *aureus* cells and monitored the bacterial burden at the wound site. At 24 hours post infection (hpi), *S*. *aureus* CFU were significantly reduced when co-infected with *E*. *coli* as compared to single species *S*. *aureus* infection (**Fig 6A**). Upon co-infection with *S*. *aureus* and *E*. *coli* Δ*pks*, *S*. *aureus* CFU remained similar to *S*. *aureus* single species infected wounds (**Fig 6B**), whereas co-infection *E*. *coli* Δ*barA* or *E*. *coli* Δ*uvrY* resulted in increased *S*. *aureus* survival, but not restoration to single species levels (**Fig 6C**), similar to our *in vitro* results (**Fig 3A**). Together, these data demonstrate that both the *E*. *coli pks* island and the BarA-UvrY TCS are important for the growth antagonism of *S*. *aureus* observed in mixed species infections *in vivo*.

## Discussion

*Escherichia coli* and *Staphylococcus aureus* are both important pathogens that cause wound infections, blood infections, urinary tract infections and infective endocarditis [55–57]. Both *E*. *coli* and *S*. *aureus* can exhibit polymicrobial synergy with other bacterial species during infection, which is advantageous for these pathogens, but often leads to adverse disease outcomes [7, 9, 25]. While many studies have investigated the mechanistic basis of polymicrobial interactions between different microbial species, the molecular interactions between *E*. *coli* and *S*. *aureus* have not been reported. In this study, we found that *E*. *coli* production of colibactin is responsible for the growth antagonism toward *S*. *aureus*, resulting in significant inhibition of *S*. *aureus in vitro* and *in vivo* during polymicrobial wound infection. *E*. *coli pks* genes are upregulated during co-culture with *S*. *aureus*, supporting the proposed role of *E*. *coli* colibactin as an effector for niche adaptation or domination [58]. Finally, we found that the *E*. *coli* two component signal transduction system BarA-UvrY senses the polymicrobial environment leading to the upregulation of the *pks* island via the Csr system.

While colibactin is best studied for its genotoxicity toward eukaryotic cells, a functional role for the *pks* island in polymicrobial interactions has also been reported. Colibactin altered the gut microbiome composition in newborn mice when the pregnant mothers were previously colonized with *pks*^+^ *E*. *coli*, with Firmicute reduction observed starting 35 days after birth [58]. Colibactin killing was also demonstrated in *Vibrio cholera*, with an interesting suggestion that the colibactin susceptibility of specific bacterial species could be related to the efficiency of endogenous DNA repair mechanisms [59]. More relevant to our work, *E*. *coli* episomally expressing the *pks* island spotted onto lawns of *S*. *aureus* gave rise to small zones of inhibition around the *pks*^+^ *E*. *coli* colonies, although they did not confirm that colibactin was the factor producing antibiotic activity [60]. We similarly observed that this antagonism was more efficient within macrocolonies, where *E*. *coli* completely inhibits the growth of *S*. *aureus* within 24 hours as compared to planktonic cultures, where we only saw significant growth inhibition at 48 hours. These data indicate that colibactin-mediated growth inhibition of *S*. *aureus* is favored at proximity but is not a biofilm dependent phenotype.

The ClbS resistance protein, which hydrolyzes colibactin into a non-toxic compound, protects the mammalian host DNA from colibactin-mediated damage [42, 61] and confers to *S*. *aureus* full protection from *E*. *coli*-mediated growth inhibition. Therefore, we conclude that mature colibactin, rather than a colibactin intermediate, is the factor inhibiting the growth of *S*. *aureus*. Consistent with this conclusion is the observation that the most efficient growth inhibition of *S*. *aureus* occurred when both *E*. *coli* and *S*. *aureus* were in close contact within a macrocolony biofilm, whereas killing was less efficient in planktonic co-culture, which would be expected for a highly unstable molecule such as colibactin [36]. Moreover, while NMDA impaired the integrity of the *S*. *aureus* membrane and has been shown to modestly inhibit *B*. *subtilis* growth [45], this colibactin intermediate is not responsible for *S*. *aureus* killing by *E*. *coli*. To date, it is not known how colibactin enters mammalian or bacterial target cells; however, the ability of NMDA to compromise the membranes of *S*. *aureus* could serve as a mechanism for colibactin entry in some circumstances.

This study, along with a contemporaneous report [48], is the first to show that *pks* island genes are regulated by the BarA-UvrY TCS system, extending *pks* regulatory inputs beyond iron limitation [46, 47]. One of the direct targets of the BarA-UvrY TCS is the Csr system, which in turn regulates diverse functional pathways such as glycolysis, gluconeogenesis, and expression of virulence factors such as biofilm formation, toxin production and pilus expression [50]. BarA-UvrY activation induces the expression of *csrB* and *csrC* [62, 63] which negatively regulate the activity of the CsrA transcriptional regulator [50]. Here we show that CsrA regulates the expression of the *pks* island via interactions with the mRNA of the *pks* regulator ClbR. CsrA have also been shown to bind to the mRNA of ClbQ, an enzyme involved in colibactin synthesis, suggesting that CsrA may also regulate the expression of other *pks* island genes by direct binding of their mRNA [48]. Together, our data support a link between the BarA-UvrY TCS, the Csr system, and colibactin synthesis. Colibactin function has largely been studied in the context of colorectal cancer [36]. Since we know that *pks* is regulated by both iron and the BarA-UvrY system, and since iron may not always be a limited nutrient in the gastrointestinal (GI) tract [64], these facts suggest that BarA-UvrY may be the predominant regulator of *pks* island expression in the GI tract. Short-chain fatty acids (SCFAs) such as acetate, propionate and butyrate are abundant in the GI tract [65]. Furthermore, these SCFAs have been demonstrated to be the stimulus of the BarA histidine kinase [66]. As such, the presence of SCFA could serve as a signal for upregulation of the *pks* island via the BarA-UvrY TCS in the GI tract. Consistent with a role for SCFA sensing by BarA, both *E*. *coli* and *S*. *aureus* have been reported to accumulate and increase production of these molecules when is iron is limited, which is suggested by gene expression profiles of mixed species macrocolonies [67, 68]. Overall, these results not only bridge the knowledge gap in understanding the polymicrobial interactions between *E*. *coli* and *S*. *aureus*, but also contribute to the understanding *pks* island regulation in the context of polymicrobial infections.

*E*. *coli* and *S*. *aureus* are causative agents of wound infections [69], where they can be co-isolated from polymicrobial wound infections [20, 70]. In one study, *E*. *coli* and *S*. *aureus* comprised 6.8% of the observed polymicrobial infections in wounds, second only to *P*. *aeruginosa* and *S*. *aureus* co-infections, suggesting that these species may interact during infection [71]. Our report that *pks*+ strains of *E*. *coli* inhibit *S*. *aureus* growth in wound infections suggest several possibilities. It is possible that when *E*. *coli* and *S*. *aureus* are co-isolated from wounds, the *E*. *coli* strains do not encode or express the *pks* island. Alternatively, *pks*+ *E*. *coli* that are co-isolated with *S*. *aureus* from wound infections may retain spatial segregation within wound biofilms such that colibactin is not in close enough proximity to *S*. *aureus* to severely limit its growth. It is also possible that host-dependent factors may serve to inactivate colibactin in some individuals. More detailed epidemiological, metagenomic, and pangenomic studies of wound infection microbiota are required to understand the ecological landscape within wound infections.

**Fig 7:**
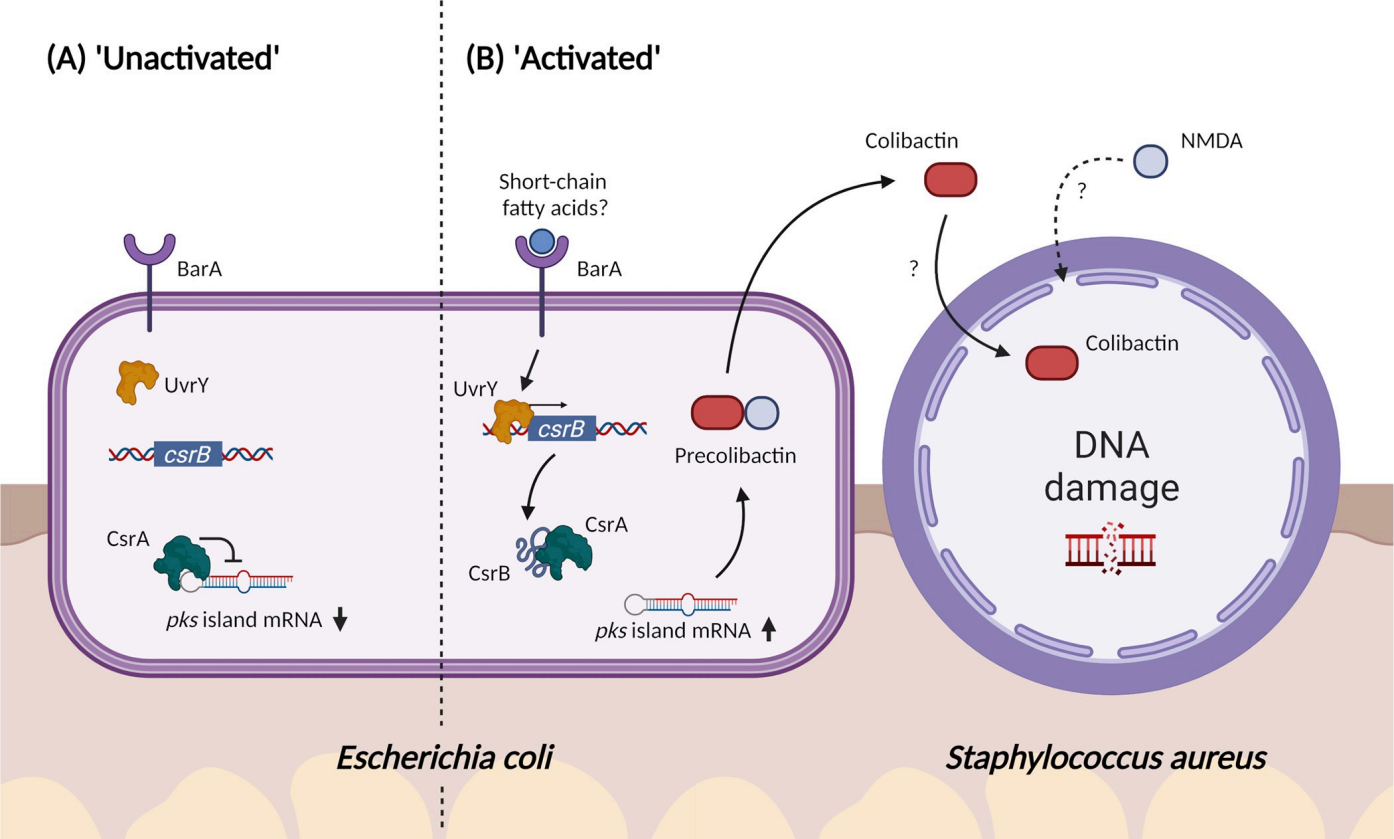
Proposed model of *E*. *coli* and *S*. *aureus* polymicrobial interactions. (A) In the absence of a signal, the BarA-UvrY two component system (TCS) is inactive. CsrA regulates the *pks* island negatively by binding the mRNA of ClbR, leading to the downregulation of *pks* island genes. (B) When *E*. *coli* and *S*. *aureus* are in proximity, the BarA-UvrY TCS is activated by the presence of specific signals, such as short-chained fatty acids. Activation of the TCS leads to upregulated expression of sRNA CsrB, which is a negative regulator of CsrA. CsrB binds to CsrA and relieves the suppression on the *pks* island, resulting in the upregulation of *pks* island genes and increased synthesis of pre-colibactin. Maturation of pre-colibactin leads to the release of both NMDA and colibactin into the environment. Entry of colibactin into *S*. *aureus*, which possibly occurs through NMDA-mediated membrane disruptions, ultimately leads to DNA damage and growth inhibition of *S*. *aureus*. The exact mechanism of colibactin entry into *S*. *aureus* remains to be determined. Created with BioRender.com.

In summary, in this study, we have shown that co-infection with *S*. *aureus* induces *E*. *coli* colibactin production, which in turn is inhibitory to *S*. *aureus in vitro* and *in vivo*, informing the microbial ecology at play during polymicrobial wound infections (**Fig 7**). Additionally, we report that the BarA-UvrY TCS indirectly regulates *pks* gene expression via the Csr system during interspecies competition. The antimicrobial spectrum of colibactin is not limited to *S*. *aureus* as previously reported [60], but extends to all the Staphylococcal species we tested. While it will be useful to have a greater understanding of the range of bacterial species that is targeted by colibactin, current knowledge raises the possibility of colibactin-related compounds as a narrow-spectrum anti-bacterial therapeutic. Its genotoxicity toward mammalian cells notwithstanding, colibactin has also been enigmatic to purify at useful yields [36], complicating its optimization as a therapeutic. Nonetheless, this work underscores the importance of the BarA-UvrY two component system in the regulation of the *pks* island, which could potentially be a therapeutic target to inhibit colibactin synthesis.

## Material and methods

### Ethics statement

All animal experiments were performed with approval from the Institutional Animal Care and Use Committee (IACUC) in Nanyang Technological University, School of Biological Sciences under protocol ARF-SBS/NIE-A19061.

### Bacteria strains and growth conditions

All strains and plasmids used in this study are listed in **Table 1**. Both *E*. *coli* and *S*. *aureus* were grown in Tryptic Soy Broth (TSB; BD Bacto, USA) at 37°C either with shaking at 200 RPM or under static conditions to late stationary phase. Overnight cultures were normalized to 1–2 x 10^8^ colony forming units (CFU)/ mL by washing the cell pellets twice with phosphate buffered saline (PBS) and then normalized to optical density (OD_600nm_) of 0.4 (*E*. *coli*) and 0.5 (*S*. *aureus*) by diluting in PBS.

**Table 1:** List of bacterial strains and plasmids used in this study.

Bacterial Strains	Description	References
***Escherichia coli***		
UTI89	Uropathogenic clinical isolate	[28]
CFT073	Uropathogenic clinical isolate	[72]
MG1655	*E*. *coli* K-12 strain, LPS mutant	[73]
Nissle 1917	Non-pathogenic gut isolate	[74]
UTI89 Δ*pks*	*pks* island knockout mutant	This study
UTI89 Δ*barA*	*barA* knockout mutant	This study
UTI89 Δ*uvrY*	*uvrY* knockout mutant	This study
UTI89-pCsrA	UTI89 (pTrc99a-CsrA)	This study
UTI89-pCsrB	UTI89(pTrc99a-CsrB)	This study
UTI89 ClbR^mut^	UTI89 strain with GGA bases mutated in *clbR* gene	This study
*E*. *coli* clinical isolate no. 6	*pks* positive clinical isolate from Tan Tok Seng Hospital	This study
*E*. *coli* clinical isolate no. 37	*pks* negative clinical isolate from Tan Tok Seng Hospital	This study
***Staphylococcus aureus***		
RN001	*S*. *aureus rsbU* mutant	[33]
HG001	RN001 derivative, *rsbU* repaired	[75]
RN6734	8325–4 derivative, *agr*^+^	[76]
MN8	Clinical isolate of toxic shock syndrome	[77]
USA300 LAC	Community-associated MRSA USA300	[78]
JE2	USA300 LAC, p01 and p03 cured	[79]
RN450	Prophage cured *S*. *aureus* strain 8325	[33]
USA300 LAC-GFP	USA300 LAC, pALC1420 GFP+	[80]
RN4220	*S*. *aureus* strain NCTC 8325–4, *sau1*^-^ *hsdR*^-^, restriction deficient	[81]
USA300-ClbS	USA300 LAC (pJC-2343-ClbS)	This study
**Plasmids**		
pKM208	Red recombinase expressing plasmid; Amp^R^	[82]
pSLC-217	P_*rhaB*_ *relE* cassette; Neo^R^	[83]
pTrc99a	*E*. *coli* cloning vector containing IPTC inducible promoter P*trc*; Amp^R^	[84]
pJC1213	*S*. *aureus* vector pT181 replicon, CmR	[85]
pJC2343	pJC1213, P*sarA* P1 promoter, CmR	This study
pTrc99a-CsrA	*E*. *coli* cloning vector pTrc99a::*csrA* (NcoI/HindIII); Amp^R^	This study
pTrc99a-CsrB	*E*. *coli* cloning vector pTrc99a::*csrB* (NcoI/HindIII); Amp^R^	This study
pJC-2343	*S*. *aureus* cloning vector containing P*sarA* P1 promoter; Cm^R^	This study
pJC-2343-ClbS	*S*. *aureus* cloning vector pJC-2343::*clbS*; Cm^R^	This study

### Generation of mutants

*E*. *coli* UTI89 mutants were generated using the positive-negative selection system as described previously [83]. Briefly, the first recombination requires amplification of the positive-negative selection cassette (Kan_RelE) from the plasmid pSLC-217 via PCR. Primers contained 50 bp homology sequence that upstream or downstream to the target gene. *E*. *coli* UTI89 carrying the pKM208 plasmid were induced with 1 mM IPTG and made electro-competent. The competent cells were transformed with 1 μg of PCR product via electroporation. The electroporated cells were recovered in LB at 37°C for 3 hr with shaking, followed by static incubation for 1 hr. The transformed cells were plated on LB agar plates supplemented with 50 μg/mL kanamycin to select for cells with the Kan_RelE selection cassette inserted into the target gene. The second recombination requires the amplification of 500 bp of DNA sequence that is upstream and downstream of the target gene and stitching them together. For the second recombination, *E*. *coli* UTI89 with the positive-negative cassette inserted into the target gene and carrying the pKM208 plasmid were induced with 1 mM IPTG and made electro-competent. The electrocompetent cells were transformed with 1 μg of the stitching fragment via electroporation. After recovery, the cells were plated on M9 agar plates supplemented with 0.2% rhamnose. The resulting knockout mutants were confirmed by colony PCR (see **S5 Table** for primers used in this study). To generate the *E*. *coli clbR*^mut^ mutant strain, the first recombination was done by amplifying of the positive-negative selection cassette (Kan_RelE) using primers containing 50 bp homology sequence that is upstream or downstream of *clbR*. The second recombination requires the amplification of the insert using primers containing 50 bp homology sequence that is upstream or downstream of *clbR*. This insert was ordered as a gBlock from Integrated DNA Technologies Pte. Ltd, Singapore. The gBlock sequence can be found in **S7 Fig**. Bases for the third GGA motif was left unmodified to avoid alteration of the ClbR protein sequence.

### Generation of plasmids

To create the ClbS expression vector, plasmid pJC-2343 was linearized with primers (InFusion_Vector_F/InFusion_Vector_R) and ClbS was amplified with primers (InFusion_ClbS_F/ InFusion_ClbS_R) using *E*. *coli* UTI89 genomic DNA as a template. PCR was performed using Q5 High-Fidelity DNA polymerase (New England Biolabs, United States) according to the manufacturer’s protocol. Thereafter, PCR purification was performed using Wizard SV Gel and PCR Clean-Up System (Promega, United States) following the manufacturer’s protocol. ClbS DNA was inserted into the linearized plasmid using In-Fusion HD Cloning system (Takara, Japan). The infusion product was used to transform into Stellar Competent Cells (Takara, Japan). The vector was linearized by inverse PCR with outward directed primers (SodA_RBS_F/ SodA_RBS_R) containing the SodA RBS and re-ligated using Kinase, Ligase, DpnI (KLD) mix (New England Biolabs, United States) [86]. The plasmid pJC-2343-ClbS was extracted from Stellar competent cells using the Monarch Plasmid Miniprep Kit and used to transform *S*. *aureus* USA300 LAC via phage generalized transduction. The *sarA* P1 promoter was amplified from NCTC 8325 with primers JCO 1141 + JCO 1142 and cloned into pJC1213 at SphI and PstI to generate pJC2343 [85]. Primers used are shown in **S5 Table**. Plasmids were verified by sequencing. Successful expression of ClbS in *S*. *aureus* USA300 LAC was verified by Western blot using guinea pig polyclonal antisera against ClbS and an anti-Guinea pig-HRP secondary antibody (Invitrogen, United States) for detection.

To create vectors for expression of *csrA* and *csrB*, the respective genes were amplified using *E*. *coli* UTI89 genomic DNA as a template and primers containing NcoI and HindIII restriction sites. PCR was performed using Q5 High-Fidelity DNA polymerase (New England Biolabs, United States) according to the manufacturer’s protocol, using primers (OEcsrA_F/OEcsrA_R) for the *csrA* insert and primers (OEcsrB_F/ OEcsrB_R) for the *csrB* insert. PCR purification was performed using Wizard SV Gel and PCR Clean-Up System (Promega, United States) in accordance with the manufacturer’s protocol. Thereafter, the vector pTrc99A and the PCR products were digested with NcoI-HF and HindIII-HF (New England Biolabs, United States) according to the manufacturer’s protocol. The vector and insert were ligated with T4 DNA ligase (New England Biolabs, United States) following the manufacturer’s protocol. The ligated product was used to transform Stellar Competent Cells (Takara, Japan). The plasmids pTrc99A-CsrA and pTrc99A-CsrB were extracted from Stellar competent cells using the Monarch Plasmid Miniprep Kit and used to transform electrocompetent *E*. *coli* UTI89. Plasmids were verified by sequencing. Primers used in this study are listed below in **S5 Table**.

### Generation of polyclonal antisera

Recombinant protein fragments were designed, expressed, and purified using the Protein Production Platform (NTU, Singapore) as previously described [87]. The ClbS target comprised of amino acid residues 2 to 166 from NCBI RefSeq accession no. ABE07674.1 and were cloned into pNIC28-Bsa4 with an N-terminal His tag followed by a TEV protease cleavage site. Polyclonal antisera were generated commercially (SABio, Singapore) by immunization of guinea pigs with purified recombinant ClbS. Specificity of the immune sera was confirmed by the absence of signal on Western blots of whole-cell lysates from wild-type *S*. *aureus* USA300 LAC with vector control.

### Macrocolony biofilm assay

*E*. *coli* and *S*. *aureus* were grown to late stationary phase and normalized as described above. Normalized cultures of *E*. *coli* and *S*. *aureus* were mixed at a 1:1 ratio for mixed species macrocolony inocula or diluted twice with PBS for single species macrocolony inocula. 5 μL of each mixture were spotted on TSB supplemented with 1.5% (w/v) agar. Macrocolonies were grown at 37°C to the required timepoint. Thereafter, the macrocolonies were harvested using a sterile blade and resuspended in PBS. For enumeration of viable CFU of each strain, the resuspension was plated on medium to select for *E*. *coli* (MacConkey; BD BBL, USA) or *S*. *aureus* (TSB supplemented with colistin and nalidixic acid; 5 μg/mL each).

### Planktonic co-culture assay

*E*. *coli* and *S*. *aureus* were grown to late stationary phase and normalized as described above. Normalized cultures of *E*. *coli* and *S*. *aureus* were mixed at a 1:1 ratio for mixed cultures or diluted twice with PBS for single cultures. 5 μL of each mixture was inoculated in 5 mL of TSB broth and grown at 37°C with shaking at 200 RPM. At specific timepoints, 200 μL of the culture was sampled for enumeration of viable CFU before performing serial dilution and plating on selective medium to select for *E*. *coli* and *S*. *aureus*.

### RNA extraction from macrocolonies

Single species and mixed species macrocolonies were grown for 6 hours followed by RNA extraction. The macrocolony was first resuspended in TRIzol Reagent (Ambion) and physical cell lysis was performed using Lysing Matrix B (MP Biomedicals). Thereafter, nucleic acids were purified via chloroform extraction followed by isopropanol precipitation. To remove DNA, DNase treatment was performed using the TURBO DNA-free kit (Ambion, USA). Ribosomal RNA (rRNA) was depleted from the samples using the RIBO-Zero Magnetic Bacterial Kit (Epicentre). RNA was converted to cDNA using the NEBNext RNA First Strand Synthesis Module and NEBNext Ultra Directional RNA Second Strand Synthesis Module (New England Biolabs, USA). Library preparation was performed by the SCELSE sequencing facility and sequenced via Illumina Miseq2500 machine as 250 bp paired reads.

### Transcriptomic analysis

RNA sequencing reads were trimmed via BBMap tools (Bushnell, 2016). The trimmed reads were mapped to *S*. *aureus* HG001 (GenBank assembly accession GCA_000013425.1) and *E*. *coli* UTI89 reference genome (GenBank assembly accession GCA_000013265.1) using BWA (version 0.7.15-r1140) [88, 89]. Reads were mapped to predicted open reading frames to each reference genome using HTSeq [90]. Gene expression analyses were done in R (version 3.4.4) using Bioconductor package, *edgeR* [91]. Gene expression differences were considered significant if the false discovery rate (FDR) was below 0.05. Annotation of genes was done using Kyoto Encyclopedia of Genes and Genomes (KEGG). The raw and processed data for the RNA-seq can be found under the GEO accession number: GSE190571.

### Generation of *E*. *coli* transposon mutant library

*E*. *coli* UTI89 were made electrocompetent, achieving a transformation efficiency of 10^7^−10^9^ CFU/μg of DNA. Briefly, pre-warmed SB medium (Tryptone, 30 g/L; yeast extract, 20 g/L; MOPS, 10 g/L) were inoculated with overnight cultures at a 1:250 ratio and incubated at 37°C with shaking at 200 RPM to mid-log phase (OD_600nm_ 0.8–0.9). The cultures were then chilled on ice for 15 min before washing the cell pellets 3 times in ice cold 10% glycerol. The cell pellets were resuspended in 1 mL of 10% glycerol and aliquoted into 50 μL aliquots. The aliquots were flash frozen in liquid nitrogen and stored at -80°C. A transposon library of *E*. *coli* UTI89 was generated with the EZ-Tn5 <R6Kγori/KAN-2>Tnp Transposome Kit (Epicentre), according to the manufacturer’s protocol. Following transformation, the electroporated cells were allowed to recover at 37°C for 1 hr in SOC media (Yeast extract, 5 g/L; Tryptone, 20 g/L; 10 mM NaCl; 2.5 mM KCl; 10 mM MgCl_2_; 10 mM MgSO_4_; and 20 mM glucose). Finally, the electroporated cells were diluted in PBS to achieve approximately 100 CFU/plate. The diluted cells were spread on Miller’s LB 1.5% (w/v) agar plates supplemented with 50 μg/mL kanamycin and incubated overnight at 37°C.

### Transposon library screen

Individual mutants of the *E*. *coli* UTI89 transposon library were inoculated in 200 μL of LB media in 96-well plates and incubated at 37°C statically overnight. A *S*. *aureus* USA300 LAC-GFP overnight culture was normalized as described above and diluted 100-fold to a final volume of 200 μL in 96-well plates before 3 μL of each UTI89 mutant were transferred into each well. Finally, 3 μL of the mixed cultures were spotted onto TSB agar and incubated at 37°C for 48 hours. A primary screen was conducted based on fluorescence intensity within the macrocolony, indicative of viable GFP-expressing *S*. *aureus*. Subsequently, mutants from the primary screen were validated by macrocolony biofilm assays and growth kinetic assays before whole genome sequencing was performed to identify the location of the transposon.

### Solid phase synthesis of N-myristoyl-D-Asn synthesis

The synthesis of *N*-myristoyl-D-Asn (NMDA) was performed as previously described [45], with modifications. All the solvents and reagents were purchased from commercial suppliers and used without further purification. N2-Fmoc-N4-trityl-D-asparagine [Fmoc-D-Asn(Trt)-OH], 2-chlorotrityl chloride resin (1.0mmol/g, 100~200mesh, 1%DVB) and PyBOP were purchased from GL Biochem (Shanghai) Ltd. 1H NMR was recorded on a Bruker 400 MHz spectrometer at 298 K. All chemical shifts were quoted in ppm and coupling constants were measured in Hz. Electrospray ionization mass spectrum (ESI-MS) of NMDA was measured in negative mode on a Thermo LTQ XL system.

#### Pre-activation of 2-chlorotrityl chloride resin

A polystyrene resin carrying a 2-chlorotrityl chloride linker (500 mg, 0.75 mmol, 1.5 mmol/g) was placed into a 50 mL polypropylene syringe fitted with a polyethylene porous frit (20 μm). The resin was swollen with dry DMF (3 x 10 mL). After removal of DMF, a solution of thionyl chloride (200 μL, 7.0 μmol) in DMF (5 mL) was added and the reaction mixture stirred for 1 h. The re-activated 2-chlorotrityl chloride resin (**S8A Fig**) was washed with DMF (3 x 10 mL) and dry dichloromethane (DCM, 3 x 10 mL).

#### Loading of Fmoc-D-Asn(Trt)-OH on 2-chlorotrityl chloride resin

Fmoc-D-Asn(Trt)-OH (**S8B Fig**, 3 equiv.) was mixed with 2-chlorotrityl chloride resin (**S8A Fig**) in anhydrous DCM (10 mL), followed by addition of N,N-diisopropylethylamine (DIPEA, 3 equiv.). The mixture was shaken for 30 min at room temperature. The resin was washed with DMF (10 mL) and the remaining reactive chloride groups were quenched with a solution of DCM:MeOH:DIPEA (5 mL, 80:15:5), followed by washing with DMF (3 × 5 mL) to yield the resin (**S8C Fig**).

#### Fmoc deprotection

To the resin (**S8C Fig**, 0.75mmol) pre-swollen in DCM was added 20% piperidine in DMF (10 mL) and the reaction mixture was shaken for 10 min. The solution was drained, and the resin was washed with DMF (x3), DCM (x3). This procedure was repeated twice to obtain the resin (**S8D Fig**). Myristic acid (**S8E Fig**, 3 equiv.) and PyBOP (6 equiv.) were dissolved in DMF/DCM (50/50). DIPEA (8 equiv.) was added to the mixture to activate the carboxylic acid. The solution was added to the resin (**S8D Fig**) and the mixture was shaken for 1 h at room temperature. Completion of the coupling reaction was checked using the Ninhydrin test. The solution was drained, and the resin was washed with DMF (3 times), DCM (3 times) successively to give the resin (**S8F Fig**).

#### Cleavage of NMDA from the resin

To the resin (**S8F Fig**) was added the cleavage mixture TFA/H_2_O/TIS (95%/2.5%/2.5%, 5 mL) and the mixture was shaken for 3 h at room temperature. The resin was removed by filtration and the resin was washed with the cleavage mixture once (2.5 mL). To the combined filtrate was added dropwise cold diethyl ether to precipitate the crude NMDA. The precipitate was collected after centrifugation and the diethyl ether decanted. This solid was washed with cold diethyl ether three times (20–30 mL x3) using the centrifugation procedure. The crude product was purified by semi-preparative reverse-phase-HPLC. Semi-preparative RP-HPLC was preformed using a Shimadzu HPLC system equipped with a Phenomenex jupiter-C18 RP column (10 × 250 mm, 5 μm) with a flow rate of 2.5 mL per minute, eluting using a gradient of buffer B (90% acetonitrile, 10% H_2_O, 0.045% TFA) in buffer A (H_2_O, 0.045% TFA). The combined pure NMDA fractions after HPLC purification were lyophilized to afford *N*-myristoyl-D-asparagine in powder form.

#### Compound characterization

The obtained pure compound was characterized by 1H NMR (**S9 Fig**). (400 MHz DMSO-d6): 12.46 (br,1H COOH), 7.96 (d, J = 8 Hz,1H, C(O)NHCH), 7.32 (s, 1H, C(O)NH2), 6.87 (s, 1H, C(O)NH2), 4.47–4.51 (m, 1H,NHCH), 2.52 (dd, J = 5.7, 15.5 Hz, 1H, CH2C(O)NH2), 2.41 (dd, J = 7.2, 15.5 Hz, 1H,CH2C(O)NH2), 2.07 (t, J = 7.3 Hz, 2H, C(O)CH2CH2),1.48–1.42 (m, 2H, C(O)CH2CH2), 1.28–1.19 (m, 20H, myristoyl-CH2), 0.85 (t, J = 6.8 Hz, 3H, CH2CH3). ESI-MS: m/z [M–H]^-^calculated for C18H33N2O4^-^ 341.24 (isotopic), observed 341.39 (**S10 Fig**).

### *N*-myristoyl-D-Asn growth inhibition assay

Overnight cultures of *S*. *aureus* were normalized to OD_600nm_ of 0.4 and diluted 100-fold. Thereafter, 8 μL of the diluted cultures were inoculated into 96-well plates containing 200 μL of TSB media supplemented with NMDA at 100 μM, 300 μM and 600 μM. For all tested NMDA concentrations and the vehicle control, DMSO was supplemented to 1% (v/v). The plates were incubated at 37°C in a Tecan Infinite M200 Pro spectrophotometer. Absorbance readings at 600 nm were taken every 15 min for 12 hours.

### Cell permeability assay

Cell permeability was determined using propidium iodide (PI) staining and flow cytometry. *S*. *aureus* USA300 LAC overnight cultures were sub-cultured into fresh TSB media and allowed to grow to mid-log phase at OD_600nm_ of 0.6. Subsequently, the cultures were treated with DMSO (1%), 100 μM palmitoleic acid and 600 μM NMDA and incubated at 37°C for 15 min. After the treatment, the cells were washed twice with PBS and stained with PI Buffer (Abcam) for 30 min at room temperature. Flow cytometry was performed with flow cytometer Fortessa X to identify the population of *S*. *aureus* stained positive for PI after treatment with compounds. Data analysis was performed using FlowJo, version 10.

### RNA extraction and real time quantitative PCR (RT-qPCR)

Macrocolonies were grown as described above. RNA from macrocolonies was extracted using the RNeasy Mini Kit (Qiagen, United States) according to the manufacturer’s protocol. Genomic DNA was removed by DNase treatment (TURBO DNA-free Kit, Ambion). RNA and DNA were quantified using Qubit RNA Assay Kit and Qubit dsDNA HS Assay kits (Invitrogen, United States). RNA quality was analyzed using Agilent RNA ScreenTape (Agilent Technologies, United States). RNA samples with minimum RIN value of 7.5 and DNA contamination of not more than 10% were converted to cDNA using SuperScript III First-strand Synthesis Supermix (Invitrogen, United States) with accordance to the manufacturer’s protocol. RT-qPCR reaction mix was prepared using KAPA SYBR FAST qPCR Kit Master Mix (2X) Universal (Kapa biosystem, United States) and ran on a StepOnePlus Real-Time PCR System (Applied Biosystems, USA). Primers GyrA_F/GyrA_R were used to amplify *gyrA* (Housekeeping gene), primers ClbA_F/ClbA_R were used to amplify *clbA*, primers ClbB_F/ClbB_R were used to amplify *clbB*. Primers are found in **S6 Table**.

### Mouse model of polymicrobial wound infection

Bacteria was grown as described above, normalized to 10^6^ CFU/10 μL, and used to infect wounds of C57BL/6 mice (Male, 7–8 weeks old; InVivos, Singapore) as previously described [92]. Briefly, the animals were anesthetized with 3% isoflurane. Dorsal hair was shaven and fine hair was removed after the application of Nair cream (Church and Dwight Co, Charles Ewing Boulevard, USA) and shaved using a scalpel blade. The skin was disinfected with 70% ethanol and a full-thickness wound was created with a 6 mm biopsy punch (Integra Miltex, New York, USA). The wounds were inoculated with 10 μL of the respective inoculum (*E*. *coli*, 1–2 x 10^6^ CFU; *S*. *aureus* 1–2 x 10^6^ CFU; Mixed 1–2 x 10^6^ CFU each). Thereafter, the wound site was sealed with a transparent dressing (Tegaderm 3M, St Paul Minnesota, USA). At the indicated timepoints, mice were euthanized, and the wounds were excised and homogenized in 1 mL PBS. Viable bacteria in the wound homogenates were enumerated by plating onto selective media for *E*. *coli* (MacConkey; Merck Singapore) and *S*. *aureus* (MRSA*Select* II Agar; Biorad USA).

## Supporting information

S1 Fig *E*. *coli* CFU are unchanged upon growth with *S*. *aureus*. **(A)** Enumeration of *E*. *coli* UTI89 grown alone or co-cultured with *S*. *aureus* USA300 in macrocolonies for 0, 6, 8, 16, 24 and 48 hours. N = 3 independent experiments. **(B)** Enumeration of *E*. *coli* UTI89 grown alone or together with different strains of *S*. *aureus* in macrocolonies for 24 h. N = 6 independent experiments. **(C)** Enumeration of indicated strains of *E*. *coli* from single species or mixed species macrocolonies containing *S*. *aureus*, at 24 hours. N = 6 independent biological experiments. **(D)** Enumeration of *E*. *coli* UTI89 from single species or mixed species macrocolonies co-cultured with indicated Staphylococcal species for 24 hours. N = 6 independent experiments. **(E)** Enumeration of *E*. *coli* strains UTI89 or MG1655 after planktonic growth alone or mixed with *S*. *aureus* for 24 or 48 hours. N = 6 independent experiments (**B-E**) Data from single species macrocolonies or planktonic cultures are indicated with open bars, and data from mixed species (all inoculated at a ratio of 1_EC_:1_SA_) macrocolonies are indicated with checked bars. Individual data points from each biological replicate are indicated with closed circles. No statistical significance was detected for any of the comparisons shown. Error bars represent SD from the mean.
(TIFF)

S2 Fig UTI89 *pks*, *barA* and *uvrY* mutants grow similarly. Overnight cultures of wild type and mutant *E*. *coli* strains were normalized to OD_600nm_ of 0.4 and diluted 100-fold. Thereafter, 8 μL of the diluted cultures were inoculated into 96-well plates containing 200 μL of TSB. The plates were incubated at 37°C in a Tecan Infinite M200 Pro spectrophotometer. Absorbance readings at 600 nm were taken every 15 min for 12 hours.
(TIFF)

S3 Fig Presence of *pks* in *E*. *coli* wound isolates correlate with *S*. *aureus* killing. Enumeration of *S*. *aureus* USA300 LAC mixed (1_EC_:1_SA_) macrocolonies with *E*. *coli* UTI89, UTI89 Δ*pks* and 10 of each *pks*+ and *pks*- clinical isolates. N = 1 experiment.
(TIF)

S4 Fig *N*-myristoyl-D-Asn compromises *S*. *aureus* membrane integrity. **(A)** Growth curves of WT *S*. *aureus* USA300 LAC grown in TSB supplemented with 100 μM, 300 μM and 600 μM of NMDA. An equal concentration of DMSO (1%) was used as the vehicle control. Each data point represents the mean measurement from 3 biological replicates, each the average of 4 technical replicates. Statistical analysis was done using Kruskal-Wallis Test with Dunn’s post-test to correct for multiple comparisons. **p< 0.01. **(B)** Percentage of PI positive *S*. *aureus* cells after treatment with 1% DMSO, 100 μM palmitoleic acid (16:9) or 600 μM NMDA. Statistical significance was determined by One-way ANOVA with Dunnett’s test for multiple comparison. ****p< 0.0001. Error bars represent SD from the mean.
(TIFF)

S5 Fig Supplementation with iron or iron chelator does not affect *E*. *coli*-mediated killing of *S*. *aureus*. Enumeration of *S*. *aureus* USA300 LAC and *E*. *coli* UTI89 after co-culture planktonic growth supplemented with 100 μM or 300 μM of FeCl_3_ or 50 μM of 100 μM of iron chelator 22D for 48 hours. N = 3 independent experiments.
(TIFF)

S6 Fig *E*. *coli* CFU are not affected by the presence of *S*. *aureus* during wound infection. Mice were infected with *E*. *coli* UTI89 or mutants alone, or co-infected with *E*. *coli* UTI89 and *S*. *aureus* USA300 LAC at 1–2 x 10^6^ CFU/wound. Wound CFU were enumerated at 24 h post infection. Single species infection or co-infection with **(A)** *E*. *coli* UTI89 WT, **(B)** *E*. *coli* pks mutant, or **(C)** *E*. *coli barA* and *uvrY* TCS mutants. Each black circle represents one mouse, horizontal lines represent the median. N = 2 independent experiments, each with 5–6 mice per group.
(TIFF)

S7 Fig Sequences of GGA bases mutated in *E*. *coli clbR*^mut^. (TIF)

S8 Fig NMDA synthesis scheme. (TIF)

S9 Fig 1H-NMR spectrum of *N*-myristoyl-D-Asn (recorded in DMSo-*d6* at 400 MHz). (TIF)

S10 Fig ESI spectrum of *N*-myristoyl-D-Asn (recorded in negative mode). (TIF)

S1 Table RNA-Seq results of *E*. *coli* UTI89 single macrocolony vs mixed macrocolony. (XLSX)

S2 Table RNA-Seq results of *S*. *aureus* HG001 single macrocolony vs mixed macrocolony. (XLSX)

S3 Table List of Transposon Mutants Identified from Transposon Library Screen. (XLSX)

S4 Table List of *E*. *coli* clinical isolates. (XLSX)

S5 Table List of primers. (XLSX)

S6 Table List of primers used in RT-qPCR. (XLSX)

S1 Text Supplementary materials and methods. (DOCX)
