# Supplementary figures and images for: Escherichia coli BarA-UvrY regulates the pks island and kills Staphylococci via the genotoxin colibactin during interspecies competition
Source: PLoS Pathog. 2022 Sep 6;18(9):e1010766. doi: 10.1371/journal.ppat.1010766 (PMC9481169; doi:10.1371/journal.ppat.1010766)

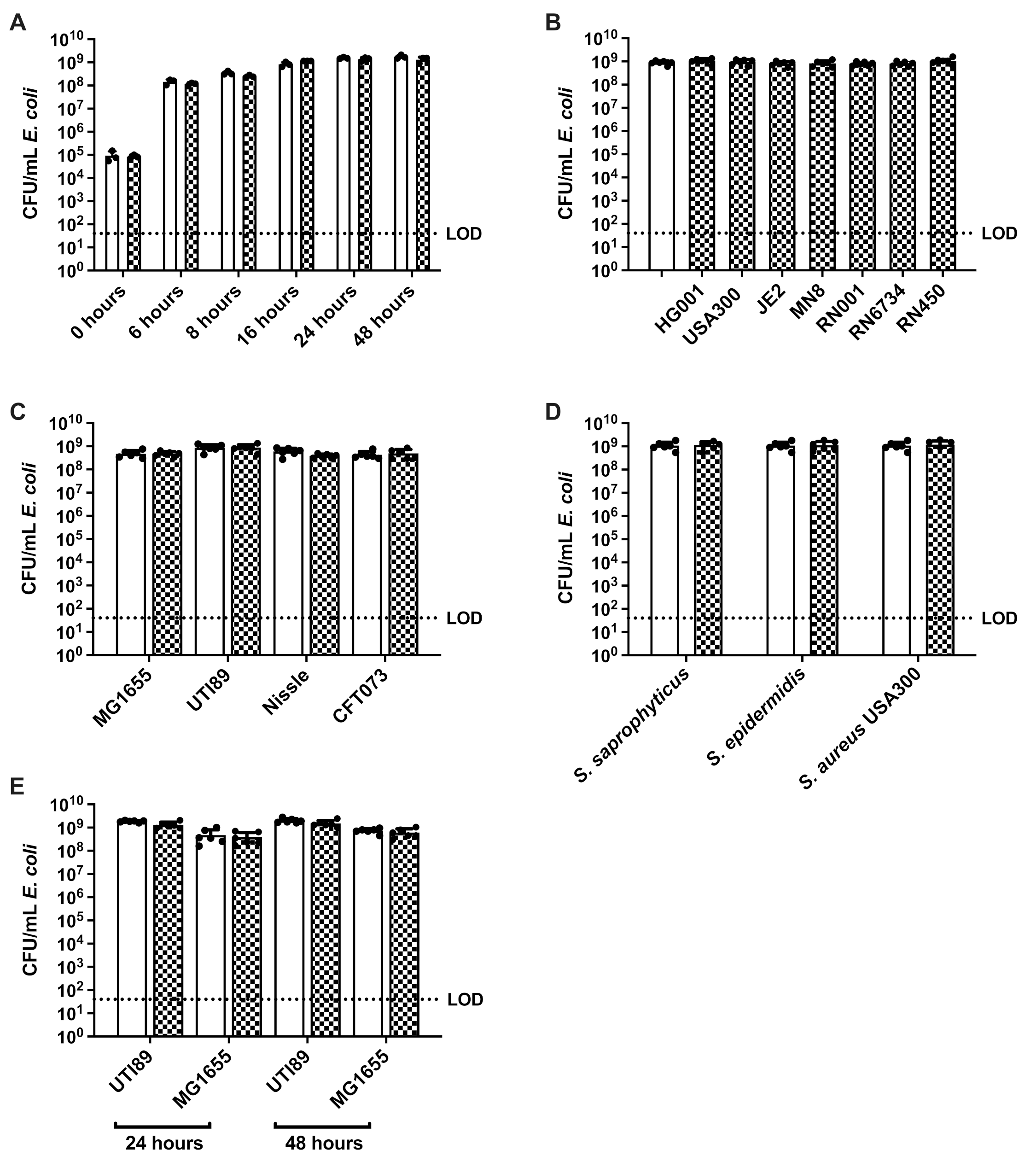

Supplement: S1 Fig — (A) Enumeration of E. coli UTI89 grown alone or co-cultured with S. aureus USA300 in macrocolonies for 0, 6, 8, 16, 24 and 48 hours. N = 3 independent experiments. (B) Enumeration of E. coli UTI89 grown alone or together with different strains of S. aureus in macrocolonies for 24 h. N = 6 independent experiments. (C) Enumeration of indicated strains of E. coli from single species or mixed species macrocolonies containing S. aureus, at 24 hours. N = 6 independent biological experiments. (D) Enumeration of E. coli UTI89 from single species or mixed species macrocolonies co-cultured with indicated Staphylococcal species for 24 hours. N = 6 independent experiments. (E) Enumeration of E. coli strains UTI89 or MG1655 after planktonic growth alone or mixed with S. aureus for 24 or 48 hours. N = 6 independent experiments (B-E) Data from single species macrocolonies or planktonic cultures are indicated with open bars, and data from mixed species (all inoculated at a ratio of 1EC:1SA) macrocolonies are indicated with checked bars. Individual data points from each biological replicate are indicated with closed circles. No statistical significance was detected for any of the comparisons shown. Error bars represent SD from the mean. (TIFF) [file ppat.1010766.s001.tiff]

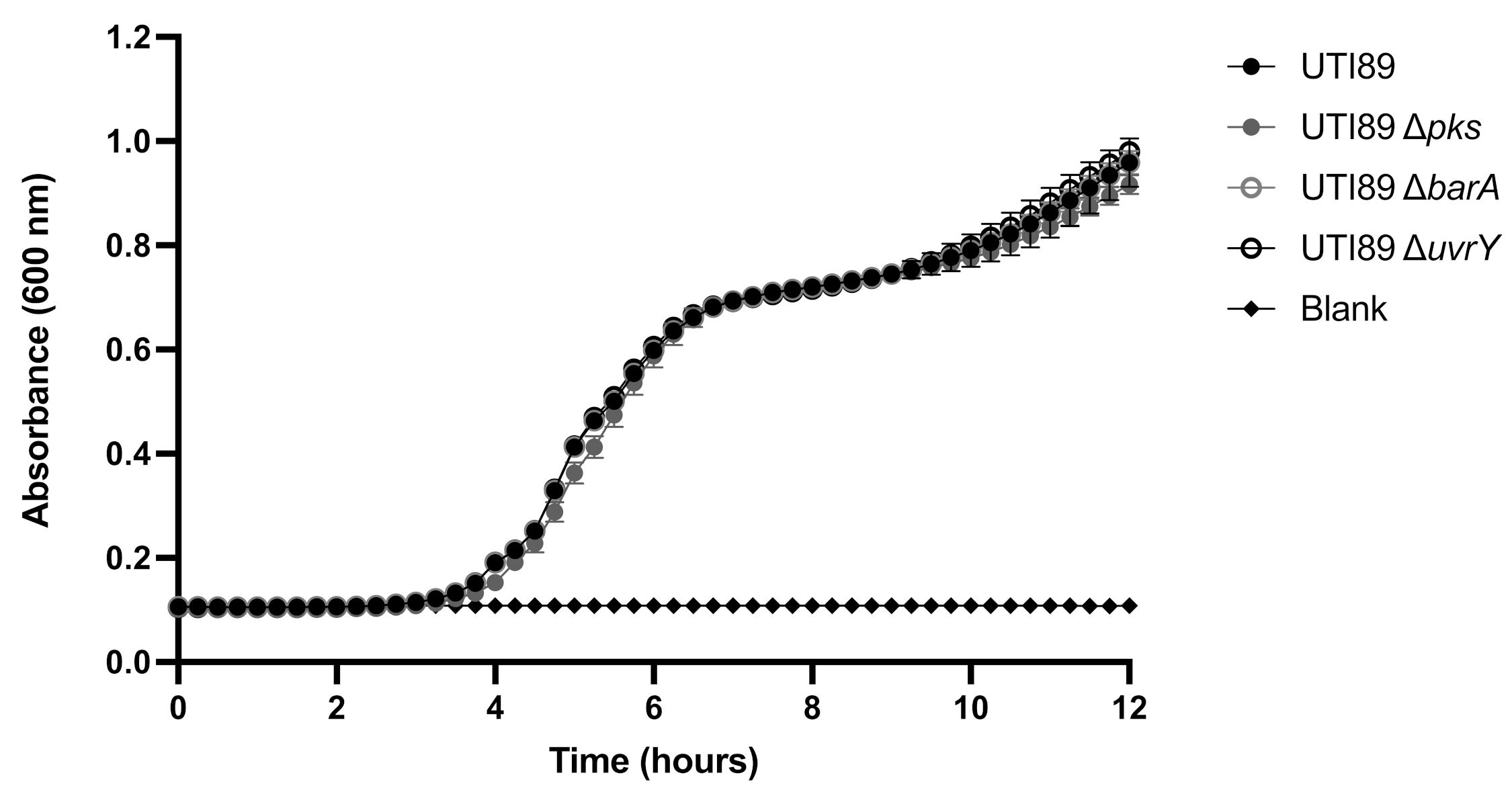

Supplement: S2 Fig — Overnight cultures of wild type and mutant E. coli strains were normalized to OD600nm of 0.4 and diluted 100-fold. Thereafter, 8 μL of the diluted cultures were inoculated into 96-well plates containing 200 μL of TSB. The plates were incubated at 37°C in a Tecan Infinite M200 Pro spectrophotometer. Absorbance readings at 600 nm were taken every 15 min for 12 hours. (TIFF) [file ppat.1010766.s002.tiff]

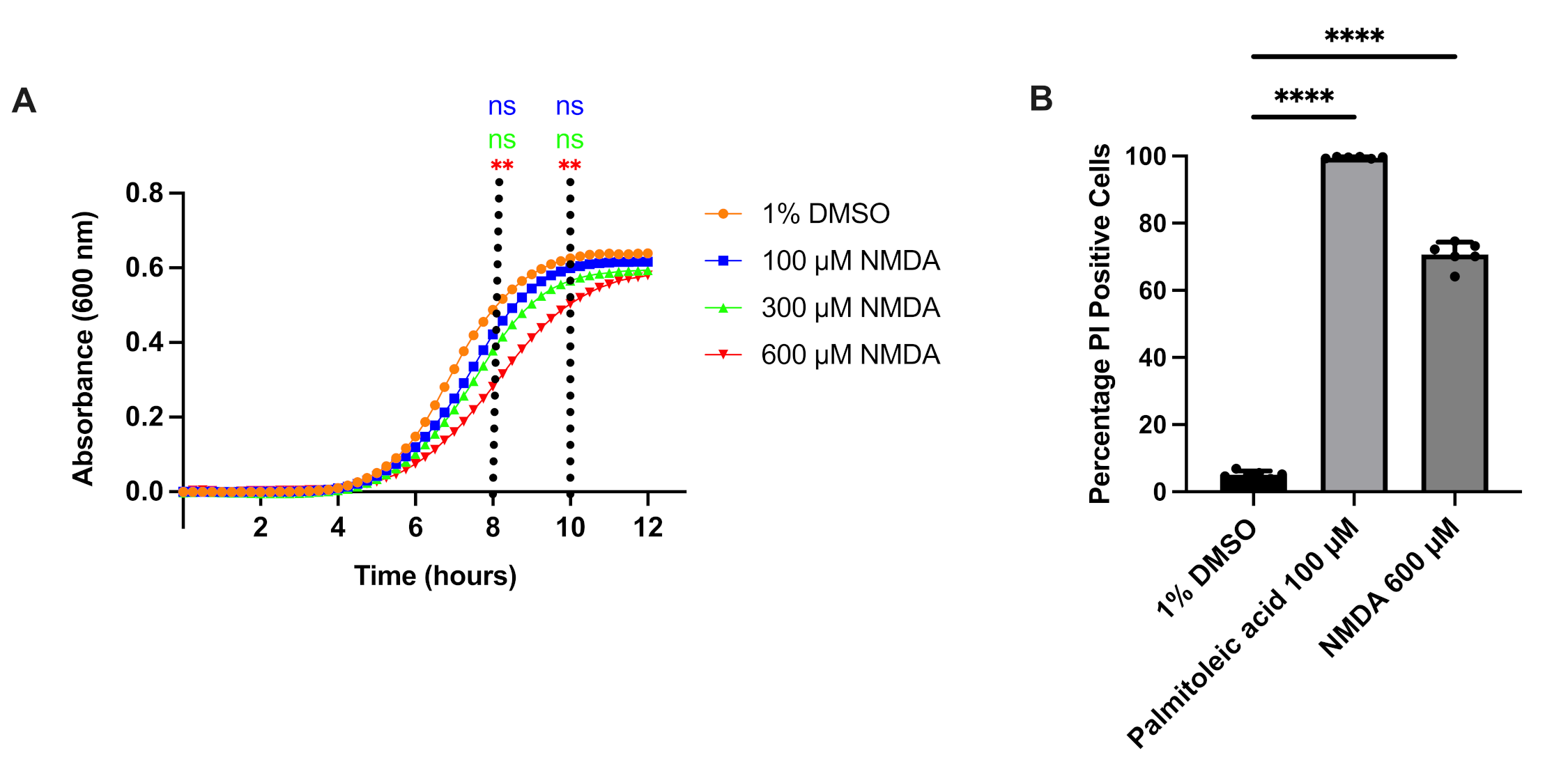

Supplement: S4 Fig — (A) Growth curves of WT S. aureus USA300 LAC grown in TSB supplemented with 100 μM, 300 μM and 600 μM of NMDA. An equal concentration of DMSO (1%) was used as the vehicle control. Each data point represents the mean measurement from 3 biological replicates, each the average of 4 technical replicates. Statistical analysis was done using Kruskal-Wallis Test with Dunn’s post-test to correct for multiple comparisons. **p< 0.01. (B) Percentage of PI positive S. aureus cells after treatment with 1% DMSO, 100 μM palmitoleic acid (16:9) or 600 μM NMDA. Statistical significance was determined by One-way ANOVA with Dunnett’s test for multiple comparison. ****p< 0.0001. Error bars represent SD from the mean. (TIFF) [file ppat.1010766.s004.tiff]

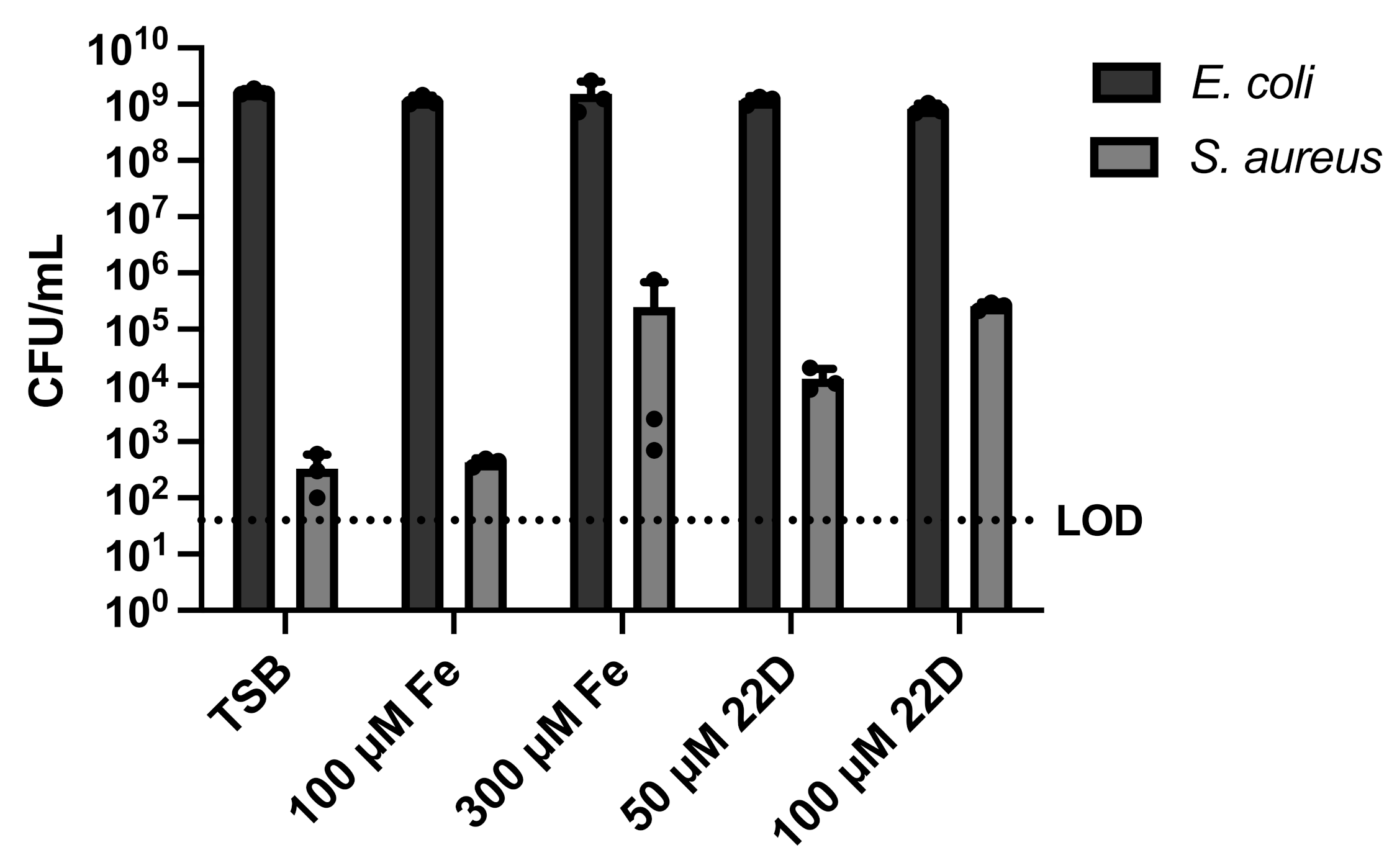

Supplement: S5 Fig — Enumeration of S. aureus USA300 LAC and E. coli UTI89 after co-culture planktonic growth supplemented with 100 μM or 300 μM of FeCl3 or 50 μM of 100 μM of iron chelator 22D for 48 hours. N = 3 independent experiments. (TIFF) [file ppat.1010766.s005.tiff]

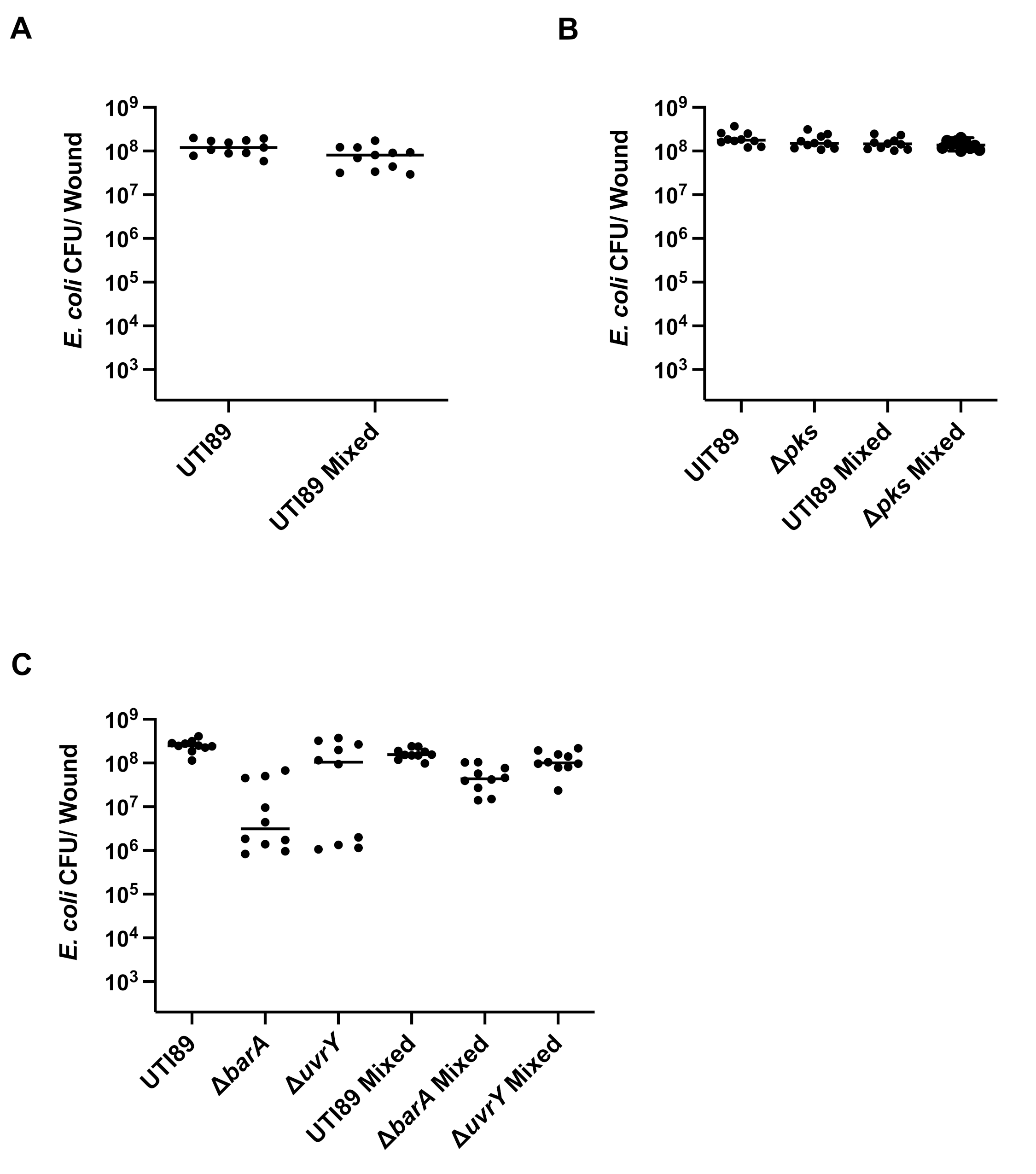

Supplement: S6 Fig — Mice were infected with E. coli UTI89 or mutants alone, or co-infected with E. coli UTI89 and S. aureus USA300 LAC at 1–2 x 10^6^ CFU/wound. Wound CFU were enumerated at 24 h post infection. Single species infection or co-infection with (A) E. coli UTI89 WT, (B) E. coli pks mutant, or (C) E. coli barA and uvrY TCS mutants. Each black circle represents one mouse, horizontal lines represent the median. N = 2 independent experiments, each with 5–6 mice per group. (TIFF) [file ppat.1010766.s006.tiff]

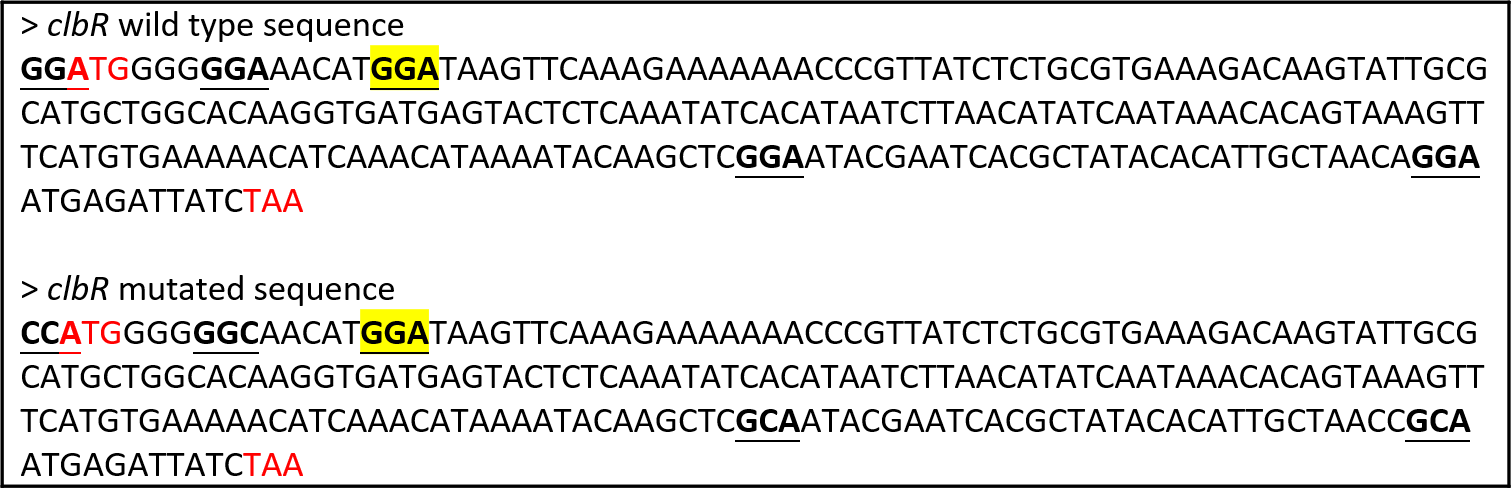

Supplement: S7 Fig — (TIF) [file ppat.1010766.s007.tif]

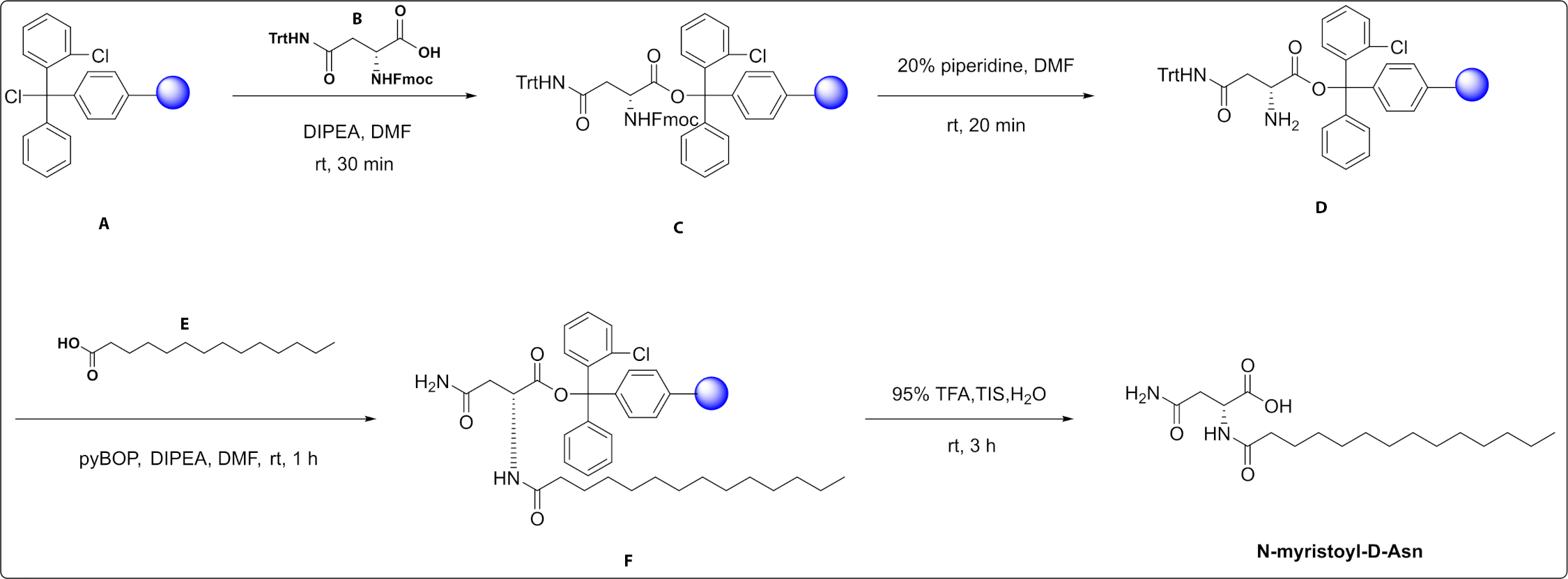

Supplement: S8 Fig — (TIF) [file ppat.1010766.s008.tif]

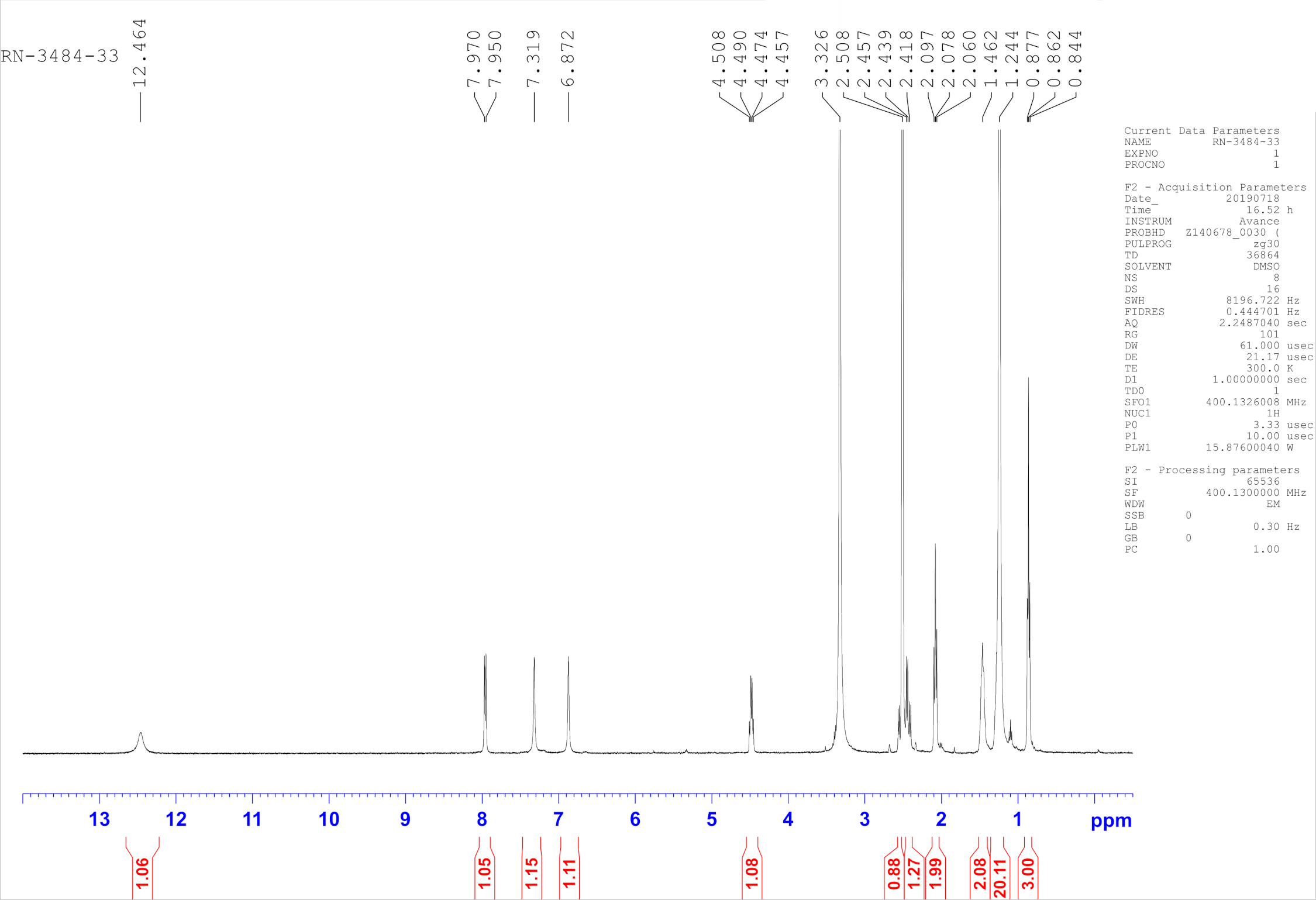

Supplement: S9 Fig — (TIF) [file ppat.1010766.s009.tif]

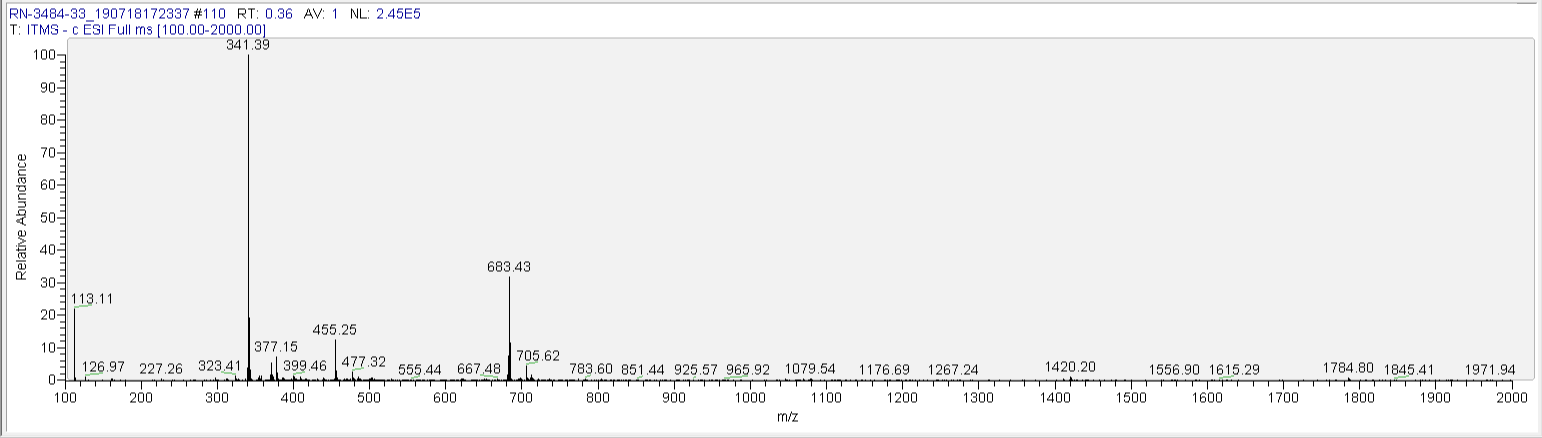

Supplement: S10 Fig — (TIF) [file ppat.1010766.s010.tif]
