# Supplementary material for: Escherichia coli BarA-UvrY regulates the pks island and kills Staphylococci via the genotoxin colibactin during interspecies competition
Source: PLoS Pathog. 2022 Sep 6;18(9):e1010766. doi: 10.1371/journal.ppat.1010766 (PMC9481169; doi:10.1371/journal.ppat.1010766)
Supplement: S1 Text — (DOCX) [file ppat.1010766.s017.docx]

**S1 Text. Supplementary Materials and Methods**

**Clinical isolate screening and macrocolony assay**

58 *E. coli* clinical isolates derived from wounds were screened for presence of *pks* by PCR using primers ClbB_F and ClbB_R and confirmed by gel electrophoresis. Wild type *E. coli* UTI89 (*pks* island containing strain) was used as a positive control while *E. coli* UTI89 Δ*pks* was used as a negative control. Upon PCR confirmation, 10 *pks* positive and 10 *pks* negative clinical isolates were selected for secondary screen by 24 hours macrocolony fluorescence assay followed by CFU enumeration (as described in the methods of the Transposon library screening section). Finally, one of each *pks* positive and *pks* negative *E. coli* clinical isolate was randomly picked to perform a 24 hours mixed macrocolony assay with *S. aureus* USA300 and USA300 pClbS strains.

**Iron and iron chelator supplementation assay**

*E. coli* UTI89 and *S. aureus* USA300 LAC were grown as described in the planktonic co-culture assay with iron supplemented into the broth at a concentration of 100 µM or 300 µM or iron chelator, 22D added at a concentration of 50 µM or 100 µM.
